# Efficacy and Safety of Tofacitinib in Patients with Polymyalgia Rheumatica (EAST PMR): An open-label randomized controlled trial

**DOI:** 10.1371/journal.pmed.1004249

**Published:** 2023-06-29

**Authors:** Xinlei Ma, Fan Yang, Jinzhi Wu, Bei Xu, Mengdi Jiang, Yiduo Sun, Chuanying Sun, Ye Yu, Danyi Xu, Lanlan Xiao, Chunyun Ren, Chunyan Chen, Zi Ye, Junyu Liang, Jin Lin, Weiqian Chen

**Affiliations:** 1 Division of Rheumatology, the First Affiliated Hospital, Zhejiang University School of Medicine, Hangzhou, Zhejiang, China; 2 State Key Laboratory for Diagnosis and Treatment of Infectious Diseases, the First Affiliated Hospital, Zhejiang University School of Medicine, Hangzhou, Zhejiang, China; 3 AnJi Branch of the First Affiliated Hospital, Zhejiang University School of Medicine, HuZhou, Zhejiang, China; 4 LinHai First People’s Hospital, TaiZhou, Zhejiang, China

## Abstract

**Background:**

Polymyalgia rheumatica (PMR) is a common inflammatory disease in elderly persons whose mechanism of pathogenesis has not been elucidated. Glucocorticoids are the main first-line treatments but result in numerous side effects. Therefore, there is a need to explore pathogenetic factors and identify possible glucocorticoid-sparing agents. We aimed to study the pathogenetic features of the disease and assess the efficacy and safety of Janus tyrosine kinase (JAK)-inhibitor tofacitinib in patients with PMR.

**Methods and findings:**

We recruited treatment-naïve PMR patients from the First Affiliated Hospital, Zhejiang University School of Medicine, between September 2020 and September 2022. In the first cohort, we found that the gene expression patterns of peripheral blood mononuclear cells (PBMCs) in 11 patients (10 female, 1 male, age 68.0 ± 8.3) with newly diagnosed PMR were significantly different from 20 healthy controls (17 female, 3 male, age 63.7 ± 9.8) by RNA sequencing. Inflammatory response and cytokine–cytokine receptor interaction were the most notable pathways affected. We observed marked increases in expression of IL6R, IL1B, IL1R1, JAK2, TLR2, TLR4, TLR8, CCR1, CR1, S100A8, S100A12, and IL17RA, which could trigger JAK signaling. Furthermore, tofacitinib suppressed the IL-6R and JAK2 expression of CD4^+^T cells from patients with PMR *in vitro*.

In the second cohort, patients with PMR were randomized and treated with tofacitinib or glucocorticoids (1/1) for 24 weeks. All PMR patients underwent clinical and laboratory examinations at 0, 4, 8, 12, 16, 20, and 24 weeks, and PMR activity disease scores (PMR-AS) were calculated. The primary endpoint was the proportion of patients with PMR-AS ≤10 at weeks 12 and 24. Secondary endpoints: PMR-AS score, c-reactive protein (CRP), and erythrocyte sedimentation rate (ESR) at weeks 12 and 24. Thirty-nine patients with newly diagnosed PMR received tofacitinib, and 37 patients received glucocorticoid. Thirty-five patients (29 female, 6 male, age 64.4 ± 8.4) and 32 patients (23 female, 9 male, age 65.3 ± 8.7) patients completed the 24-week intervention, respectively. There were no statistically significant differences in primary or secondary outcomes. At weeks 12 and 24, all patients in both groups had PMR-AS <10. PMR-AS, CRP, and ESR were all significantly decreased in both groups. No severe adverse events were observed in either group. Study limitations included the single-center study design with a short observation period.

**Conclusions:**

We found that JAK signaling was involved in the pathogenesis of PMR. Tofacitinib effectively treated patients with PMR as glucocorticoid does in this randomized, monocenter, open-label, controlled trial (ChiCTR2000038253).

**Trial registration:**

This investigator-initiated clinical trial (IIT) had been registered on the website (http://www.chictr.org.cn/, ChiCTR2000038253).

## Abstract 2

Xinlei Ma and co-workers investigate tofacitinib as a possible treatment for patients with polymyalgia rheumatica.

## Author summary

**Why was this study done?:**

- Polymyalgia rheumatica (PMR) is a common inflammatory disease in elderly persons whose pathogenesis is not elucidated. Glucocorticoids are the first-line drugs for patients with PMR but result in numerous side effects. New therapeutic agents are urgently needed.
- We investigated whether Janus tyrosine kinase (JAK) signaling was involved in the pathogenesis of PMR and if JAK-inhibitor tofacitinib was effective in the treatment of patients with PMR.

**What did the researchers do and find?:**

- In this prospective study, we showed increased expression of many genes related to JAK signaling in PMR.
- We further found that patients with PMR receiving tofacitinib (*n =* 35) or glucocorticoid (*n* = 32) both had a proportion of 100% (PMR activity disease score (PMR-AS) ≤ 10) at weeks 12 and 24. PMR-AS, c-reactive protein (CRP), and erythrocyte sedimentation rate (ESR) were all significantly decreased at weeks 12, and 24 in both groups. No severe adverse events were observed.

**What do these findings mean?:**

- These results suggest that JAK signaling is involved in the pathogenesis of PMR.
- In this study, tofacitinib monotherapy had similar benefits to glucocorticoid treatment in patients with PMR.

## Introduction

Polymyalgia rheumatica (PMR) is an inflammatory disease in elderly patients (>50 years old) characterized by pain and morning stiffness in the shoulder, neck, and pelvic girdle [1,2]. Blood tests show higher serum levels of inflammatory markers such as c-reactive protein (CRP) and erythrocyte sedimentation rate (ESR) in these patients; however, negative results of rheumatoid factor (RF), and anticitrullinated protein/peptide antibody (ACPA), antinuclear antibodies (ANA), and human leukocyte antigen B27 (HLA-B27). Sometimes, it is difficult to distinguish patients with PMR from those with seronegative late-onset rheumatoid arthritis (RA) or calcium pyrophosphate deposition (CPPD) arthropathy.

PMR is a multigene susceptibility disease, and many alleles may be involved in the pathogenesis of this disease [3]. Interleukin 6 (IL-6) polymorphisms are associated with the pathogenesis of PMR [4]. A recent paper demonstrates that IL-6 is highly expressed in the synovial tissue of 6 patients with PMR [5]. Aging is a crucial element as PMR occurs in elderly patients. Aging may increase the susceptibility to infectious agents, immune responses, or the release of cytokines [6]. However, its pathogenesis has not been fully elucidated. Patients with PMR usually have hallmark features with a dramatic response to glucocorticoids. Glucocorticoids are the first-line drugs for patients with PMR but result in numerous side effects. IL-6 receptor inhibition was reported as a therapeutic target for PMR [7]. Recently, it was shown that the IL-6 receptor inhibitor tocilizumab, in combination with glucocorticoid, is an effective therapeutic option for both new-onset PMR and glucocorticoid-dependent PMR [8–11]. Tocilizumab helps glucocorticoid tapering and even sustains glucocorticoid-free remission in patients with PMR [8]. Further pathogenetic research and the discovery of new targets can provide more ideas for the treatment of PMR. We aimed to study the pathogenetic features of PMR and assess the efficacy and safety of Janus tyrosine kinase (JAK)-inhibitor tofacitinib in patients with PMR.

## Methods

### Ethics statement

This study was approved by the Medical Ethical Committee of the First Affiliated Hospital, Zhejiang University School of Medicine (the approval number: IIT20200070C-R1). The formal consent was obtained from participant in a written informed document before the study start. The study was performed according to the recommendations of the Declaration of Helsinki.

### Study design

**Fig 1:**
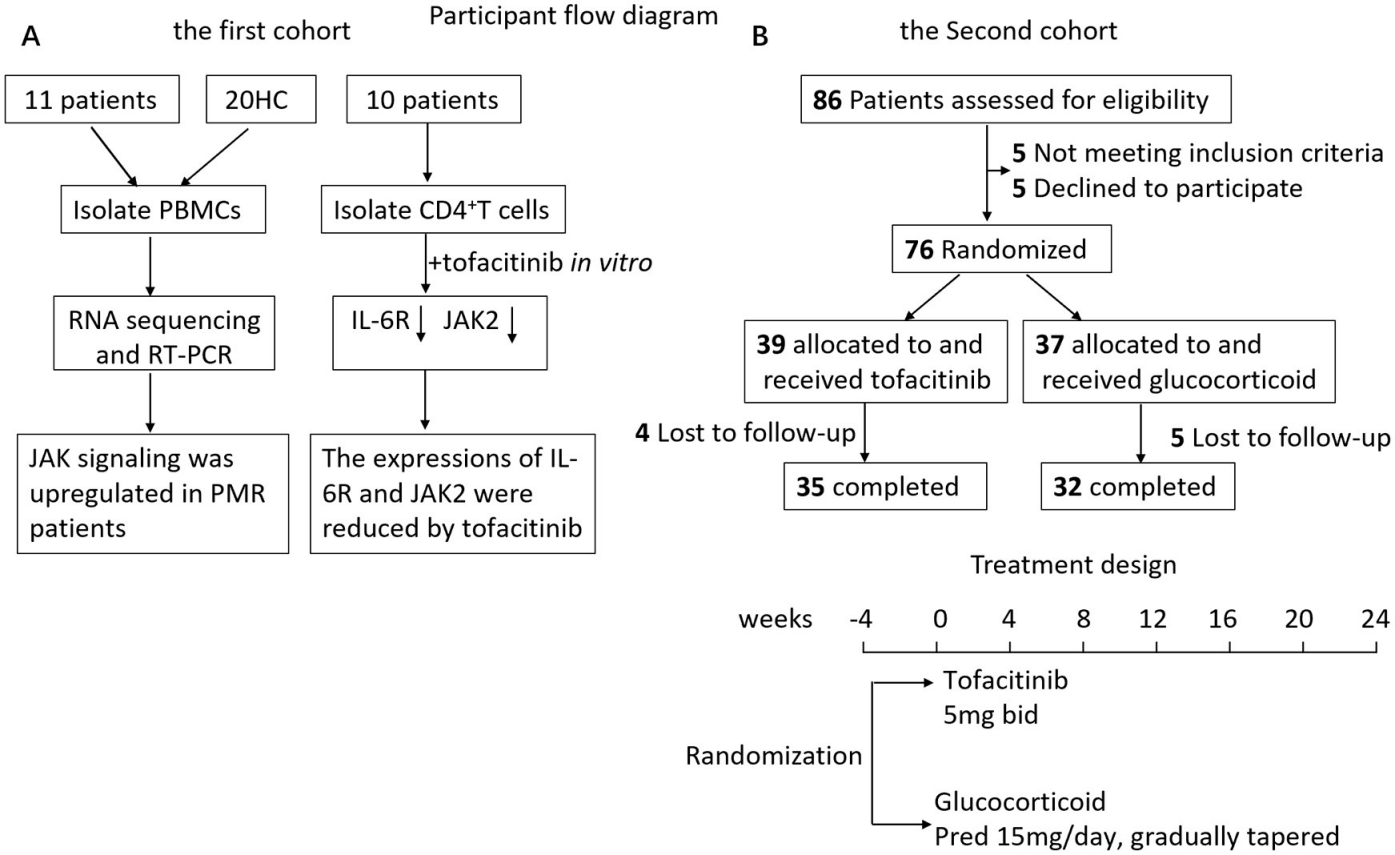
Study flowchart and treatment design. Flowchart shows recruitment, randomization, and study design. HC, healthy control; IL-6R, interleukin 6R; PBMC, peripheral blood mononuclear cell; PMR, polymyalgia rheumatica; RT-PCR, real-time PCR.

This project had 2 phases, an observational study, and a Phase II, randomized, monocenter, open-label, controlled, noninferiority trial. Trial reporting was guided by the Consolidated Standards of Reporting Trials (CONSORT) reporting guideline (S1 CONSORT Checklist). Both study designs are shown in Fig 1. This investigator-initiated clinical trial (IIT) had been registered on the website (http://www.chictr.org.cn/; ChiCTR2000038253).

### Participants

We included diagnosed PMR patients who fulfilled the 1982 Chuang criteria [12] or 2012ACR/EULAR criteria [13] between September 2020 and September 2022. Patients with high activity rheumatic polymyalgia: disease activity score PMR-AS > 10 [14] and patients with ESR > 40 mm/h or CRP > 100 mg/L (10 mg/dl) were included. They did not receive any glucocorticoids or biological agents before their inclusion in the study. There were no other connective tissue disorders, neoplasms, or current infections. In the first cohort, the pathogenetic features were studied in patients with PMR. In the second cohort, patients were treated with tofacitinib or glucocorticoids for 24 weeks.

### RNA sequence analysis and semiquantitative real-time PCR

Peripheral blood mononuclear cells (PBMCs) were isolated from 11 patients with new diagnosed PMR and 20 healthy controls (HCs) according to the protocol [15]. We also collected the PBMCs from 5 patients with PMR who were at remission under treatment with tofacitinib. Total RNA and RNA sequence libraries were prepared using an EBNext Ultra RNA Library Prep Kit for Illumina. Sequencing was performed on an Illumina NovaSeq6000. mRNA profiles were calculated with Cufflinks software and expressed as FPKM (fragments per kilobase of exon model per million mapped fragments). Some significantly altered genes were tested by semiquantitative real-time PCR. The primers for the target gene were described in S1 Table. GAPDH was considered as a normalization control. The data were examined using the 2^−ΔΔCt^ method, and results were expressed as a fold increase. Each sample was tested in triplicate, and tests were repeated 3 times.

### Isolation of CD4^+^T cells

We collect the PBMCs from patients with PMR and then isolate the CD4^+^T cells with a CD4^+^T cells isolation kit. CD4^+^T cells were cultured in soluble CD3 (1 μg/ml) and CD28 (1 μg/ml) with tofacitinib or control (DMSO). T cells were collected 24 hours later.

### Intervention, randomization, and outcomes

Patients with newly diagnosed PMR were randomized (1/1) to the tofacitinib (5 mg BID) group and the glucocorticoids (Pred 15 mg/day, gradually tapered) group using an envelope by a statistician who was not involved in the trial conduct. Rheumatologists enrolled participants, while statistician assigned participants to interventions. Statistician created 94 random numbers (from 0 to 100) using an online tool (https://random-online.com/) and put the paper with the number into each envelope without any labels. Those with odd numbers were assigned to group A and given tofacitinib, while those with even numbers were assigned to group B and given glucocorticoid. It was a block randomization. We used personalized dose-tapering schedules based on regular monitoring of patient disease activity, laboratory markers, and adverse events (AEs). Oral Prednisone 15 mg equivalent was set as the initial treatment of PMR. When improvement was achieved, the dose of Prednisone was reduced to 10 mg daily within 4 to 10 weeks. When remission was achieved, then taper Prednisone gradually by 2.5 mg every 6 to 8 weeks. Once relapse occurred, the dose increased to the pre-relapse dose. All PMR patients underwent clinical and laboratory examinations at 0, 4, 8, 12, 16, 20, and 24 weeks, and PMR activity disease scores (PMR-AS) were calculated using the previous score sheet [14]. The nonsteroidal anti-inflammatory drugs (NSAIDs) were allowed in the tofacitinib group in the first 2 weeks and then were stopped.

The primary endpoint was the proportion of patients with PMR-AS < 10 at weeks 12 and 24. Secondary endpoints: PMR-AS score, CRP, and ESR at weeks 12 and 24. Patient information on demographic data, clinical features, serological profiles, and medications was obtained from medical records.

### Statistical analysis

Results are expressed as median and standard deviation (SD). In cohort 1, comparisons of the quantitative baseline characteristics data between 11 PMR patients and 20 HCs were performed using the nonparametric Mann–Whitney test, while qualitative data were analyzed using Fisher exact test.

In cohort 2, the complete remission (CR) rate (PMR-AS < 10) at 12 and 24 weeks is the primary endpoint, and the CR rate of the patients in the experimental group is predicted as 95%, the CR rate of the control group was 85%, the margin of superiority/noninferiority was 0.1, the sample distribution ratio of the 2 groups was 1:1, calculated by PASS software, 38 cases are needed in each group. According to the 18% to 20% dropout, finally, a minimum of 47 cases per group is required. A sample size of 47 participants per group was estimated to provide at least 80% power to demonstrate the noninferiority of tofacitinib compared with glucocorticoids, with a 2-sided significance level of *P* < 0.05. We used online software to conduct power analyses. [16] In fact, at weeks 12 and 24, all patients (100%) in the tofacitinib group (*n =* 35) and glucocorticoid group (*n* = 32) had PMR-AS < 10 and had very high CR rate; therefore, we did not recruit the number of patients to 47 in each group anymore.

For the primary efficacy analysis, we included all patients who finished the follow-up. The proportion of patients with PMR-AS < 10 in 2 groups were analyzed by Fisher exact test. The secondary efficacy analysis was assessed using repeated ANOVA, including PMR-AS score, CRP, and ESR between 2 groups at weeks 0, 12, and 24. Safety was assessed for patients who were randomly assigned and received at least 1 dose of the study drug and analyzed by Fisher exact test. *P* values < 0.05 were considered statistically significant. All statistical analyses were performed using SPSS software, version 18.0.

## Results

### The patients with PMR were in a state of high inflammation

The baseline characteristics of the 11 PMR patients and 20 HCs (Fig 1) are presented in the Supporting information (S2 Table). There were no significant differences in age and sex between the PMR group and HC. In the PMR group, patients who experienced pain in the shoulder, neck, and pelvic girdle had a mean visual analogue scale (VS) score of 4.9 ± 2.2. They had higher serum levels of CRP (3.9 ± 3.1 mg/dl, normal range 0 to 0.08) and ESR (67.1 ± 24.9 mm/h, normal range 0 to 20). PMR-AS values [14] ranged from 9.33 to 27.15 with a mean value of 16.3 ± 7.3 (S2 Table), which suggests medium or high disease activity in these patients who were categorized as active PMR. They were all negative for RF, ACPA, ANA, and HLA-B27. They represented no remarkable joint swelling of wrist, metacarpophalangeal, proximal interphalangeal joint, knee, or distant joints of toes.

### Inflammatory gene expression profiles in patients with PMR

**Fig 2:**
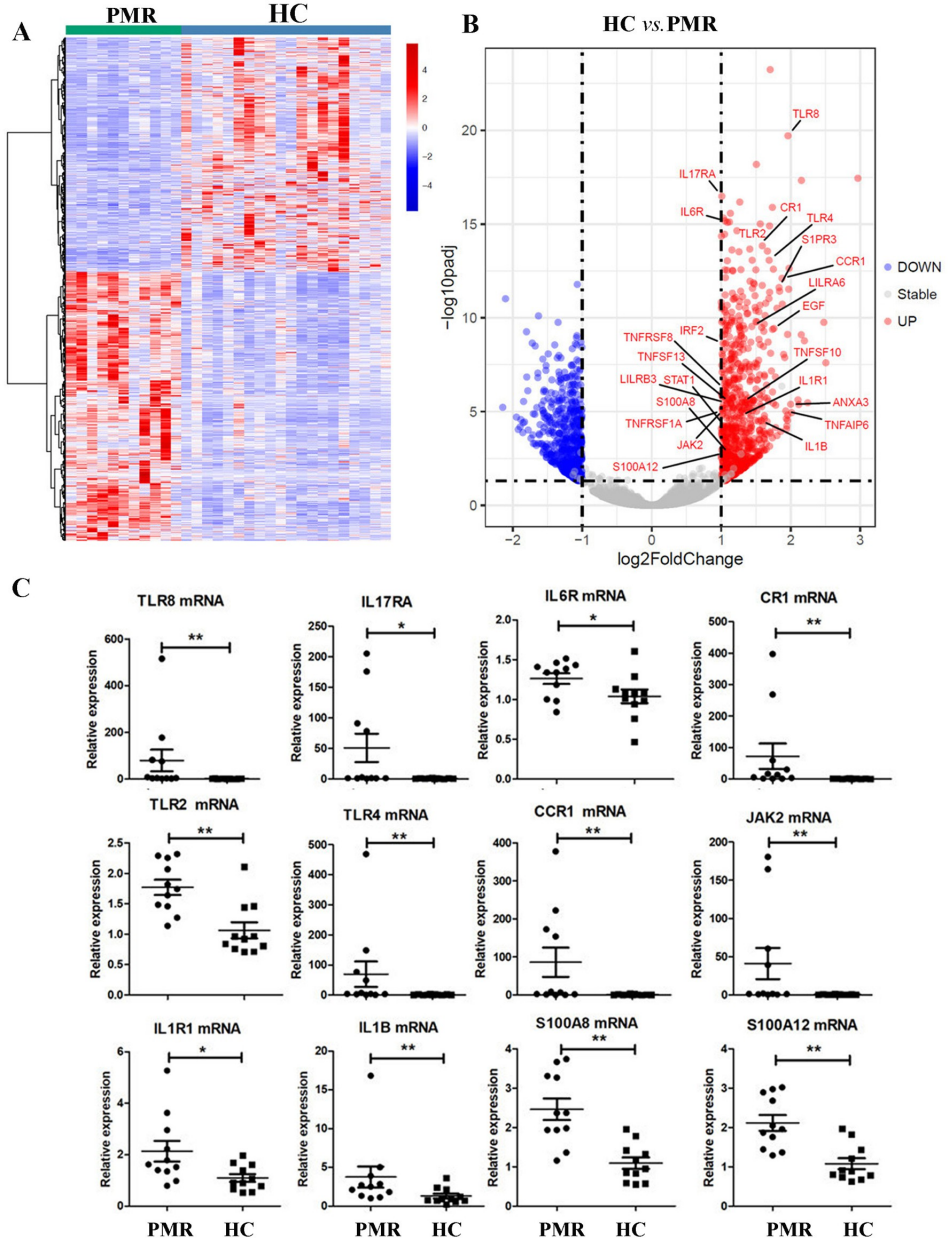
The patients with PMR had a significant inflammatory gene expression profile, abundant inflammatory genes that may activate JAK signaling. **(A)** Heatmap showed that gene expression of PBMCs was different among patients with new active PMR (*n =* 11) and HCs (*n* = 20) by RNA sequence analysis. **(B)** Volcano results demonstrated that the many genes expression were significantly increased, for example, TLR8, IL17RA, IL6R, CR1, TLR2, TLR4, CCR1, JAK2, IL1R1, IL1B, S100A8, and S100A12 (PMR *n* = 11 vs. HC *n* = 20). **(C)** They were all increased in patients with PMR by real-time PCR analysis. Comparisons between the 2 groups were performed using the nonparametric Mann–Whitney test (*p < 0.05, **p < 0.01). HC, healthy control; JAK, Janus tyrosine kinase; PBMC, peripheral blood mononuclear cell; PMR, polymyalgia rheumatica.

The principal component analysis (PCA) and heatmap showed that gene expressions of PBMCs in patients with PMR differed from those of HC (Fig 2A). There were 1,406 (758 up, 648 down) differentially expressed genes (DEGs) between PMR and HC in S3 Table. Gene Ontology (GO) analysis demonstrated that the inflammatory response (false discovery rate (FDR) 6.74 × 10^−15^, included 58 significantly altered genes), plasma membrane (7.30 × 10^−12^, included 254 significantly altered genes), integral component of the plasma membrane (5.23 × 10^−11^, included 115 significantly altered genes), extracellular space (2.76 × 10^−8^, included 103 significantly altered genes), extracellular region (4.36 × 10^−8^, included 116 significantly altered genes), response to lipopolysaccharide (2.42 × 10^−6^, included 27 significantly altered genes), integral component of membrane (1.36 × 10^−5^, included 270 significantly altered genes), immune response (1.21 × 10^−4^, included 42 significantly altered genes), cell surface (2.04 × 10^−4^, included 46 significantly altered genes), innate immune response (4.29 × 10^−4^, included 41 significantly altered genes), response to glucocorticoid (7.43 × 10^−4^, included 14 significantly altered genes), IL-1β secretion (1.67 × 10^−3^, included 6 significantly altered genes) were 12 significant predominant pathways in PMR (S1 Fig). The cytokine-mediated signaling pathway, the positive regulation of IL-6 production, and the MyD88-dependent Toll-like receptor (TLR) signaling pathway were also significantly altered. Kyoto Encyclopedia of Genes and Genomes (KEGG) analysis revealed that the cytokine−cytokine receptor interaction was the most remarkable pathway (FDR 2.85 × 10^−5^) (S1 Fig). GO and KEGG results demonstrated that patients with PMR had significant inflammatory gene expression profiles.

### Abundant inflammatory genes related to Janus tyrosine kinase (JAK) signaling in patients with PMR

Volcano results demonstrated that many genes expression were significantly increased; for example, TLR8 (*p* = 1.95 × 10^−20^), IL17RA (*p* = 3.27 × 10^−17^), IL6R (*p* = 6.71 × 10^−16^), CR1 (*p* = 1.14 × 10^−14^), TLR2 (*p* = 2.99 × 10^−14^), TLR4 (*p* = 8.48 × 10^−14^), CCR1 (*p* = 7.75 × 10^−13^), JAK2 (*p* = 6.57 × 10^−6^), IL1R1 (*p* = 1.72 × 10^−5^), IL1B (*p* = 2.87 × 10^−5^), S100A8 (*p* = 2.14 × 10^−3^), and S100A12 (*p* = 2.76 × 10^−3^) (Fig 2B). We also confirmed that the above genes were all increased via real-time PCR (Fig 2C). They were related to JAK signal transducer and activator of transcription (STAT) pathway [17–27]. Most importantly, the expression of JAK2 was increased in PBMCs from patients with PMR (Fig 2B and 2C). Therefore, JAK–STAT pathway may be crucial in the pathogenesis of PMR.

### Tofacitinib suppressed the IL-6R and JAK2 expression of CD4^+^T cells from patients with PMR *in vitro*

Next, we isolated the CD4^+^T cells from patients with PMR. CD4^+^T cells were cultured with anti-CD3 and anti-CD28 and tofacitinib or DMSO control. Tofacitinib suppressed the IL-6R and JAK2 expression of CD4^+^T cells from patients with PMR *in vitro* (S2 Fig).

### Tofacitinib effectively treats patients with PMR as glucocorticoid does

**Fig 3:**
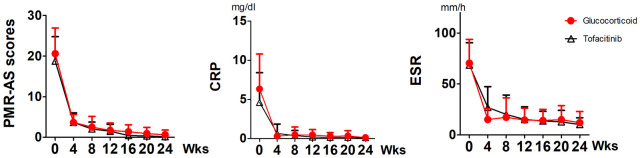
Tofacitinib effectively treats patients with PMR as glucocorticoid does. Patients with newly diagnosed PMR were randomized to the tofacitinib (5 mg BID) group and the glucocorticoids (Pred 15 mg/day, gradually tapered) group for 24 weeks. All PMR patients underwent clinical and laboratory examinations at 0, 4, 8, 12, 16, 20, and 24 weeks, and PMR-AS were calculated. At weeks 12 and 24, all patients in both groups had PMR-AS <10. PMR-AS, CRP, and ESR were all significantly decreased at weeks 12, and 24 in both groups. CRP, c-reactive protein; ESR, erythrocyte sedimentation rate; PMR, polymyalgia rheumatica; PMR-AS, PMR activity disease score.

In the second cohort, 35 patients with newly diagnosed PMR received tofacitinib and 32 patients received glucocorticoid who completed the 24-week follow-up (Fig 1). No significant difference in sex ratio and age was found between the 2 groups (Table 1). The baseline of PMR-AS was comparable in the tofacitinib (18.8 ± 6.0) and glucocorticoid group (20.6 ± 6.3, *p* = 0.18) (Table 1). At weeks 12 and 24, all patients (100%) in both groups had PMR-AS <10, so the primary outcome in 2 groups was comparable at weeks 12 and 24. We demonstrated that the confidence interval for the difference in probability between arms was 0, below the noninferiority margin 0.1; therefore, the noninferiority was established. Notably, their pain disappeared and many genes were decreased after tofacitinib therapy. The second outcomes such as PMR-AS, CRP, and ESR were all significantly decreased at weeks 12, and 24 in both groups; therefore, the second outcomes were comparable (Fig 3). PMR-AS in tofacitinib group versus glucocorticoid group was 0.16 ± 0.27 (95% confidence interval 0.049, 0.26) versus 0.62 ± 1.09 (95% confidence interval 0.16, 1.07) *p* = 0.011 at week 24. CRP was 0.13 ± 0.18 (95% confidence interval 0.06, 0.19) versus 0.15 ± 0.22 (95% confidence interval 0.067, 0.23) *p* = 0.35 at week 24. ESR was 10.09 ± 6.72 (95% confidence interval 7.71, 12.47) versus 11.89 ± 11.15 (95% confidence interval 7.94, 15.84) *p* = 0.54 at week 24. One representative case with significant gene alteration and decrease in disease activity after tofacitinib therapy is shown in S3 Fig. The mean dose of glucocorticoid was 6.3 ± 3.3 mg/day in patients in glucocorticoid arm at week 24. There were 5 patients having a relapse when the glucocorticoid tapering but quickly improved after increase in dose of glucocorticoid.

**Table 1:** Baseline characteristics of PMR patients in tofacitinib and glucocorticoid group.

	Tofacitinib (*n* = 35)	Glucocorticoid (*n* = 32)
Age, mean ± SD years	64.4 ± 8.4	65.3 ± 8.7
Female/male	29/6	23/9
Disease duration, mean ± SD months	6.4 ± 7.0	8.2 ± 15.0
Pain, VS score 0–10, mean ± SD	5.5 ± 1.3	5.4 ± 1.2
Physician global assessment, VS score 0–10, mean ± SD	5.5 ± 1.2	5.3 ± 1.3
Morning stiffness, min, mean ± SD	18.4 ± 18.1	17.0 ± 14.7
EUL, mean ± SD	1.3 ± 0.9	2.1 ± 0.9
CRP, mean ± SD mg/dl	4.7 ± 3.8	6.3 ± 4.5
ESR, mean ± SD mm/h	68.7 ± 21.7	70.7 ± 23.7
PMR-AS, mean ± SD	18.8 ± 6.0	20.6 ± 6.3
The use of NSAIDs in the first 2 weeks (%)	100	0

CRP, c-reactive protein; ESR, erythrocyte sedimentation rate; EUL, ability to elevate the upper limbs (0 = lifted above the shoulder girdle, 1 = to the shoulder girdle, 2 = below the shoulder girdle, 3 = cannot lift); NSAID, nonsteroidal anti-inflammatory drug; PMR, polymyalgia rheumatica; PMR-AS, PMR activity disease score; SD, standard deviation; VS, visual analogue scale.

PMR-AS: CRP (mg/dl) + patient self-evaluation (0–10 visual scale) + physician global assessment (0–10 visual scale) + [morning stiffness (min) × 0.1] + EUL (0–3).

Furthermore, the blood samples were collected from 4 patients under treatment with tofacitinib at week 24, and it was isolated from 1 patient at week 12. The gene expression profiles of patients with PMR that were in remission after tofacitinib therapy (the PMR-R group) differed from those of patients in the newly diagnosed PMR with active disease activity (PMR) group (PMR-R versus PMR; 1,403 DEGs shown in S4 Table) and were similar to those of HC group (PMR-R versus HC; 48 DEGs shown in S5 Table) (S4 Fig). Many genes were decreased after tofacitinib therapy. These results suggested that patients with PMR-R had attained complete serological remission after tofacitinib therapy.

In the tofacitinib group, 2 patients personally reduced the dose of tofacitinib from 5 mg BID to 5 mg QD at week 20 and then kept the dosage until week 24. Another patient reduced the dose of tofacitinib from 5 mg BID to 5 mg QD at week 8 and then kept the dosage until week 24. All 3 patients were always in remission even after reducing the dose of tofacitinib.

One patient in the tofacitinib group had a rapid decrease in PMR-AS score (28.72 to 2.85) at the initial 4 weeks, but had an increase in PMR-AS score (7.77) at week 12, finally reduced to lower disease activity at week 24. One patient in the glucocorticoid group had a good response well (24.87 to 2.2) at the initial 16 weeks, but had disease exacerbation (2.2 to 7.6) at week 20, eventually a disease score of 4.97 at week 24. There were 4 patients lost to the follow-up due to traffic inconvenience but still had a good response to tofacitinib (PMR-AS score < 10) at weeks 8 to 12. In the control group, 5 cases had a good response to glucocorticoid (PMR-AS score < 10) at weeks 8 to 16 but were lost to the follow-up after week 16.

### Safety

There were no severe AEs or death in the 2 groups (Table 2). No upper respiratory infection, tuberculosis, cardiovascular event, deep vein thrombosis (DVT), pulmonary embolism (PE), or cancer during the 6-month period. No temporary discontinuation due to AE or permanent discontinuation due to AE. There were 2 cases with mild urinary infection, 2 cases with mild herpes zoster, and 1 case with mild cytopenia in the tofacitinib group, while 1 case with new hypertension, 11 cases with hyperglycemia, 2 cases with mild cytopenia, and 2 cases with mild abnormal liver function in glucocorticoid group. One patient had a mild urinary infection after 4 weeks of treatment with tofacitinib, and another patient had a mild urinary infection after 20 weeks; they both recovered quickly to antibiotics without interruption of tofacitinib. Two patients had mild herpes zoster in tofacitinib group and recovered quickly to antiviral treatment without stopping tofacitinib. One patient had increased blood pressure after 2 weeks of treatment with glucocorticoid, falling back to normal level after additional antihypertensive therapy. One patient had mild hyperglycemia after 2 weeks of treatment of glucocorticoid, maintained at a stable level during the remaining period. There were 4 cases with new hyperlipidemia in the tofacitinib group, but 11 cases with new hyperlipidemia in the glucocorticoid group (*p* = 0.032) (Table 2).

**Table 2:** AE of tofacitinib and glucocorticoid during the 24-week period.

	Tofacitinib *n* = 39	Glucocorticoid *n =* 37	*P* value
SAE	0	0	
Death	0	0	
Urinary infection	2	0	0.26
Herpes zoster	2	0	0.26
New hypertension or deterioration	0	1	0.487
Hyperglycemia	0	1	0.487
DVT or PE	0	0	
Cancer	0	0	
Cytopenia	1	2	0.48
New hyperlipidemia	4	11	0.032
Abnormal liver function	0	2	0.234
Temporary discontinuation due to AE	0	0	
Permanent discontinuation due to AE	0	0	

AE, adverse event; ALT, alanine aminotransferase; AST, aspartate aminotransferase; DVT, deep vein thrombosis; HDL-C, high-density lipoprotein cholesterol; LDL-C, low-density lipoprotein cholesterol; PE, pulmonary embolism; SAE, severe adverse event; TC, total cholesterol; TG, triglyceride; ULN, upper limit of normal.

Cancer: new malignancy or patients with malignant tumors who have been successfully treated for more than 5 years before screening, recent recurrence; cytopenia: Hb <90 g/L, or neutrophil <1 × 10^9^, L <0.5 × 10^9^; hyperlipidia: TG >2.26 mmol/L, TC ≥6.22 mmol/L, LDL-C ≥4.1 mmol/L, HDL-C <1.0 mmol/L; abnormal liver function: ALT/AST 2 times higher than ULN.

## Discussion

PMR is a classic inflammatory disease with an acute onset but an unknown cause. In the first cohort, we found that the patients with PMR had a significant inflammatory gene expression profile related to JAK signaling via RNA sequence analysis. Furthermore, tofacitinib suppressed the IL-6R and JAK2 expression of CD4^+^T cells from patients with PMR *in vitro*. Those data suggested that JAK signaling was involved in the pathogenesis of PMR. Furthermore, in the second cohort, patients with newly diagnosed PMR were randomized to tofacitinib group and glucocorticoid group for 24 weeks. We found that similar benefits were achieved with tofacitinib monotherapy and glucocorticoid treatment in patients with PMR.

As we know, patients with active PMR or RA both commonly suffer from severe pain. During clinical practice, NSAIDs are used to relieve pain for patients with RA. Tofacitinib usually works at week 2 in the treatment of RA. The NSAIDs were allowed in the tofacitinib group in the first 2 weeks and then were stopped. We can still consider the therapeutic effect of tofacitinib for PMR during the 24-week period. Furthermore, no severe side effects were found in the 24-week period of treatment of tofacitinib. We also found that the response rate was higher as expected in patients with PMR. We think tofacitinib may have a high response rate in the new diagnosed PMR patients who were naïve to glucocorticoid or biological agents. If we treat PMR patients who were relapsing on prior glucocorticoid or MTX or other agents in the real world, the expected response rate of tofacitinib may be not high.

IL-6 is one of the key pro-inflammatory cytokines involved in the pathogenesis of PMR [5]. IL-6 and IL-1β were increased in the inflamed trapezius and lateral femoral muscles of PMR patients [17]. IL-6 can activate the JAK–STAT signaling via IL6R. Meanwhile, IL-1β binding to IL1R1 can form the high-affinity IL-1R complex, which mediates the nuclear factor kappa B (NF-κB), mitogen-activated protein kinase (MAPK), and JAK–STAT activation and promotes inflammatory response [18]. Innate immune activation is found in patients with PMR [28]. TLR2, TLR4, and TLR8 were highly expressed in peripheral blood leukocytes, particularly in monocytes and macrophages. TLRs can lead to NF-κB activation, cytokine secretion, and the inflammatory response. TLR2/TLR4 in collaboration with the JAK–STAT signaling regulates endotoxemia and inflammation [19,20]. STAT-1 was required for TLR8 transcriptional activity [21]. Furthermore, TLR4 was associated with increased susceptibility to PMR [29].

S100A8 is an inflammatory mediator that can induce the JAK1/2-dependent signaling [22]. S100A12 was induced by IL-6 via JAK signaling [23]. CCR1 and CR1 play critical roles in the process of inflammation. CCR1 was involved in the regulation of smoke-induced inflammation via JAK/STAT3 signaling [24]. CCR1 may also play a role in macrophage and endothelial cell infiltration in experimental arthritis models partly through activating JAK signaling [25]. Complement activation can be induced during inflammation, which is further normalized by a JAK1/2 inhibitor [26]. The number of Th17 cells was increased in the blood of PMR patients. CD161^+^CD4^+^T cells, which are precursors of Th17 cells, differentiated into Th17 cells through STAT-1/STAT-3 phosphorylation [27]. We found that IL6R, IL1B, IL1R1, TLR2, TLR4, TLR8, S100A8, S100A12, CCR1, CR1, and IL17RA expressions were increased in PBMCs from patients with PMR. They can activate the JAK–STAT pathway [17–27]. They were also related to the function of monocytes, T cells, and neutrophils. Most importantly, the expression of JAK2 was increased in PBMCs from patients with PMR. These genes may be partly involved in the pathogenesis of PMR via JAK–STAT signaling.

As we know, glucocorticoids are the first-line drugs in the treatment of PMR. However, they come with numerous side effects. JAK1 and JAK3 were involved in the chronic inflammation of media and large arteries in an animal model of giant cell arteritis (GCA) [30]. JAK inhibitor (JAKi) tofacitinib effectively suppressed innate and adaptive immunity in the vessel wall by reducing the proliferation rates of lesional T cells and their interferon-γ, IL-17, and IL-21 expressions [30]. To some extent, RA and PMR are 2 inflammatory diseases with similar manifestations. JAKi was used for the treatment of RA. JAKi may be one choice for PMR. Interestingly, tofacitinib, in combination with prednisone, was tried in 14 patients with PMR (11 newly diagnosed patients, 3 relapsing patients), demonstrating an efficacy of tofacitinib as a steroid-sparing agent in an open-label Phase II single-arm trial [31]. Another JAKi baricitinib lowered rapidly disease activity and exerted a significant steroid-sparing effect in 6 cases with refractory PMR and/or GCA [32]. JAKi appears as an appealing option for treating patients with PMR if they have refractory disease course or other diseases such as diabetes mellitus or osteoporosis, which is relatively contraindicated for use of glucocorticoids. Due to tofacitinib patent running out and price falling, patients from countries of lower income levels can also afford the cost of this drug.

Here, we did an IIT study to explore whether single tofacitinib effectively treats new patients with PMR as glucocorticoid does. Excitingly, patients with PMR had a good response to tofacitinib therapy or steroid therapy in our study. We also collect the safety data of the drug after 24 weeks. No severe side effects or deaths were found in the 24-week period of treatment of tofacitinib. Recent ORAL surveillance study demonstrated that RA patients aged ≥50 years with ≥1 additional cardiovascular risk factor (for example, current cigarette smoker, hypertension, diabetes mellitus) have higher risks of major adverse cardiovascular event (MACE) and cancers during a median follow-up of 4.0 years, comparing the combined tofacitinib doses (5 or 10 mg twice daily) with tumor necrosis factor inhibitors (TNFis) [33]. If patients did not have a history of atherosclerotic cardiovascular disease, MACE risk did not appear different between tofacitinib (5 mg twice daily) and TNFi group in a post hoc analysis from ORAL surveillance [34]. As we know, PMR patients also aged ≥50 years commonly have cardiovascular risk factors such as hypertension and diabetes mellitus. Therefore, if we treat the PMR patients with tofacitinib 5 mg twice daily much longer, they may have an increased cardiovascular risk as well as PMR patients who are on the long treatment of glucocorticoids [35]. However, many patients can reduce to a lower dose of tofacitinib 5 mg daily after remission. We think the cardiovascular risk may reduce when the dose of tofacitinib tapering. Even so, the cardiovascular risk of tofacitinib needs further investigation in patients with PMR at lower doses.

Our study has some limitations. The number of patients was small recruited from our medical center. We do not finish the functional experiment of candidate genes involved in the pathogenesis of PMR. PMR is closely associated with GCA, which is mainly an inflammatory vascular syndrome involving medium—and large—arteries. In most cases, PMR occurs in isolation but may be found in 40% to 60% of patients with GCA [1] and 16% to 21% of initially isolated PMR develop into GCA [29]. The clinical association and pathophysiological studies suggest that GCA and PMR represent different clinical disease spectrum in the same disease process. Subclinical GCA can be detected in patients with PMR by PET scan, but such vascular imaging is not frequently conducted in patients with likely PMR alone [36]. The possibility of obvious GCA, not subclinical GCA, may be excluded by cure with low-dose steroids. No assessment of subclinical GCA was performed in the study. Further studies are needed to investigate the clinical role and mechanism of action of candidate genes in PMR.

## Conclusions

Many inflammatory genes associated with JAK signaling are highly enriched in PBMCs from patients with PMR. Tofacitinib, a pan JAKi, effectively treated the PMR patients with clinical remission and a sharp decrease in CRP and ESR in this clinical trial study. Tofacitinib may effectively treat PMR patients. In the future, whether patients with PMR can be treated with tofacitinib alone should be carried out in the large phase randomized controlled trial.

## Supporting information

S1 CONSORT Checklist CONSORT 2010 checklist of information to include when reporting a randomized trial. (DOC)

S1 Protocol This supporting information contains the following items: (1) Original protocol in English. (2) Original protocol in Chinese. (3) Approved letter for this study in our hospital.
(PDF)

S1 Fig KEGG and GO analysis of gene expression of PBMCs from PMR and HC. (JPG)

S2 Fig Tofacitinib suppressed the IL-6R and JAK2 expression of CD4^+^T cells from patients with PMR *in vitro*. (TIF)

S3 Fig One representative case after tofacitinib therapy. (TIF)

S4 Fig The gene expression profiles of patients with PMR that were in remission after tofacitinib therapy. (TIF)

S1 Table Primers for RT-PCR. (DOCX)

S2 Table Demographic and clinical parameters of 11 patients with PMR and 20 healthy controls in the first cohort. (DOCX)

S3 Table Differentially expressed genes (DEGs) between PMR and HC. (XLSX)

S4 Table Differentially expressed genes (DEGs) between PMR and PMR-R. (XLSX)

S5 Table Differentially expressed genes (DEGs) between PMR-R and HC. (XLSX)

## Abbreviations

ACPA: anticitrullinated protein/peptide antibody
AE: adverse event
ANA: antinuclear antibody
CPPD: calcium pyrophosphate deposition
CR: complete remission
CRP: c-reactive protein
DEG: differentially expressed gene
DVT: deep vein thrombosis
ESR: erythrocyte sedimentation rate
FDR: false discovery rate
FPKM: fragments per kilobase of exon model per million mapped fragments
GCA: giant cell arteritis
GO: Gene Ontology
HC: healthy control
HLA-B27: human leukocyte antigen B27
IIT: investigator-initiated clinical trial
IL-6: interleukin 6
JAK: Janus tyrosine kinase
JAKi: JAK inhibitor
KEGG: Kyoto Encyclopedia of Genes and Genomes
MACE: major adverse cardiovascular event
MAPK: mitogen-activated protein kinase
NF-κB: nuclear factor kappa B
NSAID: nonsteroidal anti-inflammatory drug
PBMC: peripheral blood mononuclear cell
PCA: principal component analysis
PE: pulmonary embolism
PMR: polymyalgia rheumatica
PMR-AS: PMR activity disease score
RA: rheumatoid arthritis
RF: rheumatoid factor
SD: standard deviation
STAT: signal transducer and activator of transcription
TLR: Toll-like receptor
TNFi: tumor necrosis factor inhibitor
VS: visual analogue scale
