# Supplementary material for: Efficacy and Safety of Tofacitinib in Patients with Polymyalgia Rheumatica (EAST PMR): An open-label randomized controlled trial
Source: PLoS Med. 2023 Jun 29;20(6):e1004249. doi: 10.1371/journal.pmed.1004249 (PMC10309604; doi:10.1371/journal.pmed.1004249)
Supplement: S1 Protocol — This supporting information contains the following items: (1) Original protocol in English. (2) Original protocol in Chinese. (3) Approved letter for this study in our hospital. (PDF) [file pmed.1004249.s002.pdf]

This supplement contains the following items:

1. Original protocol in English

2. Original protocol in Chinese

3. Approved letter for this study in our hospital

Clinical Protocol

Efficacy and safety of tofacitinib in patients with Polymyalgia Rheumatica (EAST PMR): a prospective study

Research Institution: the First Affiliated Hospital, Zhejiang University School of Medicine

Proposal date: 19 July 2020

Principal investigator: Lin, Jin

Version date: July 19, 2020

Version number: 2.0

Protocol Revision History

**Edition**	**Date**	**Note**
1.0	2020-Jun-17	First draft
2.0	2020-July-19	Revised Draft

Investigator statement and signature page

This study is a prospective clinical study, and the methodology and drugs used in the study have been widely used clinically. This study will establish a strict adverse event monitoring system to carefully record all adverse events that occur and deal with them in a timely and effective manner. In case of any serious adverse event or important adverse event, no matter whether it is related to the research intervention or not, and no matter whether the intervention operation has been implemented or not, the research sponsor must be notified in time, and the researcher should decide whether to stop the research in a timely manner according to the situation. The researchers guarantee that the personal data of the subjects will be kept strictly confidential: all subject information and images will be identified by numbers rather than names; identifiable information will not be disclosed to members outside the research team, unless the subjects are obtained All research members and research sponsors are required to abide by the principle of confidentiality; all research files will be kept in locked filing cabinets, which are only available to researchers; during the implementation of the research and after completion, government management departments are allowed Or the members of the ethics committee will conduct spot checks and supervision on the personal data of the subjects according to the regulations; when the results of this research are published, no personal data will be disclosed.

Researcher's signature

I have read and agreed to the plan proposed in this document, and I agree with the relevant content of the plan. During the experiment, I will strictly abide by the laws and regulations of the People's Republic of China and relevant rules and regulations, perform the duties of the researcher and abide by the confidentiality regulations.

Institution ： ______________________

Name： ______________________

Position： ______________________

Signature： ______________________

Date： ______________________

Contents

Abstract **5**
Abbreviation **8**
Flowchart **9**
1. Background **12**
2. Objectives **12**
3. Study design **12**
3.1 Design 12
3.2 Randomization 13
3.3 Sample and allocation 13
4. Subjects **13**
4.1 Inclusion cretria 13
4.2 Exclusion cretria 13
4.3 Exit (loss) 14
4.4 Withdrawal 15
4.5 Termination 15
5. Treatment **15**
5.1 Study drug 15
5.2 Oral administration 15
5.3 Randomization 16
5.4 Treatment and follow up 16
6. Study procedures **16**
6.1 Screening phase 16
6.2 Follow-up 1 Weks 0 17
6.3 Follow-up 2（Weks4±3days）、3（Weks8±3days）、5（Weks 16±3）、6（Weks 20±3days） 17
6.4 Follow-up 4（Weks 12±3days）、7（Weks 24±3days） 18
7. Efficacy evaluations **19**
7.1 The primary outcomes 19
7.2 The secondary outcomes 19
7.3 Efficacy evaluations 19
8. Safety **19**
8.1 Adverse event 19
8.2 Lab test 19
9. Concomitant diseases and treatment **20**
9.1 Concomitant diseases 20
9.2 Treatment 20
9.3 Prohibited drugs 20
10. Handling of adverse event (AE) and serious adverse event (SAE) **21**
10.1 Definition of AE 21
10.2 Definition of SAE 22
10.3 Lack of efficacy 23
10.4 Abnormal clinical test results 23

10.5 How to find adverse event....24
10.6 Records of AE and SAE....24
10.7 Assessment of AE and SAE....24
10.8 Visits for AE and SAE....26
10.9 Management and Reporting of SAE....26
10.10 AE and SAE after the end of study....27
10.11 AE and SAE related to trial....27
10.12 Treatment for AE in this trial....27
11. Drugs adherence....28
12. Withdrawal....28
12.1 Subject completes the trial....28
12.2 Subject withdraws from trial....28
12.3 Screening and baseline assessment failure....29
13. Data management....29
14. Statistical analysis....30
14.1 Sample size determination....30
14.2 Analysis set....31
14.3 Statistical methods....31
14.4 Safety analyses....32
14.5 Statistical software....32
14.6 Interim analysis....32
15. Research management....32
15.1 Comply with GCP requirements....32
15.2 Privacy of subjects....32
15.3 Informed consent....33
15.4 Protocol revision procedure....33
15.5 Case report form recording and modification procedures....33
15.6 Quality Control....33
15.7 Data storage....34
15.8 Schedule....34
16. Paper publication....35
17. References....35
Table 1 Classification Criteria for Polymyalgia Rheumatica in 1982....36
Table 2 Classification Criteria for Polymyalgia Rheumatica in 2012....36
Table 3 Polymyalgia rheumatica disease activity (PMR-AS) score....36

Abstract

Title	Efficacy and safety of tofacitinib in patients with Polymyalgia Rheumatica (EAST PMR): A Randomized Clinical Trial
Disease	Polymyalgia Rheumatica
Objective	Assess the efficacy and safety of JAK-inhibitor tofacitinib in patients with PMR
Institution	the First Affiliated Hospital, Zhejiang University School of Medicine
Participants	Patients who met the inclusion criteria and were not in exclusion criteria
Sample	We will recruit 47 cases to the tofacitinib treatment group and 47 cases to the control treatment group.
Drugs	1. tofacitinib，5mg, tablet（patients pay）;<br>2. prednison，5mg, tablet（patients pay）;<br>3. Methylprednisolone，4mg tablet（patients pay）;
Dosage regimen	**Treatment group:** tofacitinib 5mg bid, 24 weeks of treatment<br><br>**Positive control group:** prednisone (or equivalent dose of methylprednisolone) 15mg/d, 2 weeks; 12.5mg/d, 2 weeks; 10mg/d, 6 weeks; 7.5mg/d, 6 weeks; 5mg/d, 8 weeks, a total of 24 weeks.<br>If the disease relapses, the steroid dose is returned to the original dose.<br><br>(If tofacitinib treatment is ineffective for 6 weeks during the follow-up, the clinician can adjust the patient to the control group. If the pain is obvious, diclofenac 75mg qd or other similar drugs are allowed. Presence of osteoporosis: calcium carbonate D3 0.6g qd)
Inclusion criteria	1. PMR patients who fulfilled the 1982 Chuang criteria (Table 1) or 2012ACR/EULAR criteria (Table 2) for PMR；They did not receive any glucocorticoids or biological agents during the three-month period that preceded their inclusion in the study;<br>2. Patients with high activity rheumatic polymyalgia: disease activity score PMR-AS (Table 3) > 10,<br>3. Adults age 50-75, Weight 45-80Kg,<br>4. ESR>40mm/h or CRP >100mg/L（10mg/dl）,<br>5. Informed consent.
exclusion criteria	1. Patients with known allergies to tofacitinib, prednisone or methylprednisolone;<br>2. Patients with giant cell arteritis, systemic lupus erythematosus, rheumatoid arthritis and other rheumatic diseases;

	3. Patients with severe osteoarthritis;<br>4. Subjects with any severe acute, chronic or recurrent infection (e.g. pneumonia or pyelonephritis, recurrent pneumonia, chronic bronchiectasis, tuberculosis, etc.);<br>5. Patients with abnormal liver function (ALT/AST 2 times higher);<br>6. Pregnant or lactating women;<br>7. Patients with a history of malignancy who do not meet the following condition (patients with malignant tumors who have been successfully treated for more than 5 years before screening without any evidence of recurrence);<br>8. Patients with previous visual field disorder or single eye dysfunction, cataract patients;<br>9. Patients with decompensated cardiac insufficiency or severe hypertension or diabetes mellitus, i.e. systolic blood pressure higher than 160mmhg or diastolic blood pressure higher than 100mmhg;<br>10. For patients with abnormal blood routine, WBC < 2 x 10^9 / L, HGB < 60g / L, or PLT < 50 x 10^9 / L, it is up to the doctor to determine whether the drug can be used;<br>11. Patients with active bleeding and peptic ulcer;<br>12. Patients with other autoimmune diseases.<br>13. Have used biological agents 6 months before enrollment;<br>14. Added azathioprine, leflunomide, methotrexate, cyclosporine (or any other calcineurin inhibitors) within 1 month before enrollment, or adjusted the dosage of the above drugs within 1 month patient;<br>15. Those who have participated in other drug clinical trials within 4 weeks.
Follow-up	Screening、Weks 0、4、8、12、16、20、24
Outcomes	Primary outcomes: Proportion of patients with PMR-AS <10 at 12 and 24 weeks<br>Secondary outcomes: PMR-AS score, CRP, ESR at 12 and 24 weeks
Safety	1. Observe the occurrence of adverse events and adverse reactions such as infection, herpes zoster, rash,<br>Diarrhea, positive stool OB;<br>2. Laboratory abnormalities found in blood and urine routine tests, liver, and kidney function, etc.;<br>3. Abnormal findings in general physical examination (such as heart rate and blood pressure);

	4. Bone density, lumbar fracture, femoral head necrosis;<br>5. Glaucoma，etc.;
Analysis	For the primary efficacy analysis: PPS analysis set;<br>For the second efficacy analysis: PPS analysis set;<br>For the safety analysis: SS analysis set.

Abbreviation

英文缩写	全称
AE	Adverse event
ALT	Alanine aminotransferase
ANA	Antinuclear antibody
APTT	Partially activated prothrombin time
AST	Aspartate aminotransferase
BUN	Blood urea nitrogen
CR	Complete remission
CRE	Creatinine
CRF	Case report form
CRP	C reactive protein
ECG	Electrocardiogram
ESR	Erythrocyte sedimentation rate
GCP	Good Clinical Practice for Drug Clinical Trials
HGB	hemoglobin
MMF	mycophenolate mofetil
NR	No reaction
PPS	Per-protocol dataset
PR	Partial remission
RBC	Red blood cell count
SAE	Serious adverse event
SS	Security set
WBC	White blood cell count

Flowchart

**Weeks of follow-up**	**Weks -4 ~0**	**Follow-up 1 Week 0**	**Follow-up 2 Weks 4±3 days**	**Follow-up 3 Weks 8±3 days**	**Follow-up 4 Weks 12±3 days**	**Follow-up 5 Weks 16±3 days**	**Follow-up 6 Weks 20±3 days**	**Follow-up 7 Weks 24±3 days**
Sign the informed consent	√							
Inclusion/Exclusion Criteria	√							
Demographics	√							
Past medical history	√							
Vital signs	√	√	√	√	√	√	√	√
Weight and height	√							
Physical examination	√	√	√	√	√	√	√	√
Disease activity indicator: PMR-AS score	√		√	√	√	√	√	√
Patient pain VAS Score	√		√	√	√	√	√	√
Physician global assessment	√		√	√	√	√	√	√
Morning stiffness time (min)	√		√	√	√	√	√	√
Difficulty in raising the upper arm 0,1,2,3	√		√	√	√	√	√	√
Difficulty in squatting and standing up 0,1,2,3	√		√	√	√	√	√	√

ESR	√		√	√	√	√	√	√
CRP	√		√	√	√	√	√	√
Blood routine [1]	√		√	√	√	√	√	√
Urine routine [2]	√		√	√	√	√	√	√
Liver function [3]	√		√	√	√	√	√	√
Renal function [4]	√		√	√	√	√	√	√
Blood glucose	√		√	√	√	√	√	√
Coagulation indicators [5]	√							√
Immunological test [6]	√							
Hepatitis B antigen and antibody, HBV-DNA	√							
T-spot	√							
Chest X-ray	√							
PET-CT (optional)	optional							optional
Shoulder and hip MRI (optional)	optional							optional
Bone mineral density (BMD)	√							optional
Electrocardiogram	√							
Echocardiography	√							
Randomization		√						

Concomitant medication	√	√	√	√	√	√	√	√
Adverse event	√	√	√	√	√	√	√	√

Note: 1. Blood routine: red blood cell count, hemoglobin, white blood cell count, platelet count, neutrophil count, lymphocyte count.

2. Urine routine: urine protein, urine red blood cells, urine white blood cells.

3. Liver function: alanine aminotransferase (ALT), aspartate aminotransferase (AST), albumin (ALB).

4. Renal function: blood urea nitrogen (BUN), creatinine (CRE).

5. Coagulation indicators: prothrombin time (PT), activated partial thromboplastin time (APTT), fibrinogen (FIB).

6. Immunological test: rheumatoid factor (RF), anti-CCP antibody (ACPA), Human leukocyte antigen B27 (HLA-B27), anti-nuclear antibody (ANA), anti-ENA antibody, anti-cardiolipin antibody, immunoglobulin and complement levels, anti-myeloperoxidase antibody (MPO), anti-proteinase 3 antibody (PR3).

Backgroud

Polymyalgia rheumatica (PMR) occurs frequently in elderly patients over 50 years old, manifested as pain in the neck, shoulder joints, scapular muscles, and pelvic girdle muscles. It is a kind of vascular inflammation that seriously affects the quality of life of patients. At present, glucocorticoids are the main drug for the treatment of PMR, which can quickly relieve pain, reduce inflammatory indicators, and improve the quality of life. However, the overall duration of glucocorticoid treatment is at least 1 year, and it is easy to relapse when the glucocorticoid is reduced; this patient group itself also has a high proportion of osteoporosis, diabetes, cardiovascular disease and other chronic diseases, and glucocorticoids can cause a variety of side effects, such as osteoporosis, peptic ulcer, steroid diabetes, glaucoma, secondary infection, metabolic disorders, etc., therefore, looking for more good therapeutic drug is an urgent clinical problem to be solved. Tofacitinib has been approved for the treatment of highly inflammatory diseases such as rheumatoid arthritis, to rapidly relieve joint symptoms and reduce inflammatory responses. There have been case reports (https://www.isr.ie/abstracts/a-review-of-patients-started-on-janus-kinase-inhibitor-jaki-in-university-hospital-waterford/) 3 patients with PMR had the symptoms improved after Tofacitinib treatment, but there is currently a lack of systematic clinical research to confirm the clinical efficacy of the drug on PMR. This topic aims to clarify the safety and effectiveness of tofacitinib on PMR. In order to observe the efficacy and safety of tofacitinib in the treatment of PMR, this clinical study is now planned.

Objectives

To assess the efficacy and safety of JAK-inhibitor tofacitinib in patients with PMR.

Study design

3.1 Design

This study was a randomized, single-center, prospective study followed the GCP principles. The whole test will be completed by the research center according to the protocol.

3.2 Randomization

The implementation of randomization uses envelopes to generate random numbers for randomized grouping.

3.3 Sample and allocation

3.3.1Sample

We will recruit 47 cases to the tofacitinib treatment group and 47 cases to the control treatment group.

3.3.2 Allocation

Patients who met the inclusion criteria and were not in exclusion criteria

Subjects

4.1 Inclusion criteria

（1） PMR patients who fulfilled the 1982 Chuang criteria (Table 1) or 2012ACR/EULAR criteria (Table 2) for PMR；They did not receive any glucocorticoids or biological agents during the three-month period that preceded their inclusion in the study,

（2） Patients with high activity rheumatic polymyalgia: disease activity score PMR-AS (Table 3) > 10,

（3） Adults age 50-75, Weight 45-80Kg,

（4） ESR>40mm/h or CRP >100mg/L (10mg/dl),

（5） Informed consent.

4.2 Exclusion criteria

（1） Patients with known allergies to tofacitinib, prednisone or methylprednisolone;

（2） Patients with giant cell arteritis, systemic lupus erythematosus, rheumatoid arthritis, and other rheumatic diseases;

（3） Patients with severe osteoarthritis;

（4） Subjects with any severe acute, chronic or recurrent infection (e.g. pneumonia or pyelonephritis, recurrent pneumonia, chronic bronchiectasis, tuberculosis, etc.);

（5） Patients with abnormal liver function (ALT/AST 2 times higher);

（6） Pregnant or lactating women;

（7） Patients with a history of malignancy who do not meet the following condition (patients with malignant tumors who have been successfully treated for more than 5 years before screening without any evidence of recurrence);

（8） Patients with previous visual field disorder or single eye dysfunction, cataract patients;

(9) Patients with decompensated cardiac insufficiency or severe hypertension or diabetes mellitus, i.e. systolic blood pressure higher than 160mmhg or diastolic blood pressure higher than 100mmhg;

(10) For patients with abnormal blood routine, WBC < 2 x 10^9 / L, HGB < 60g / L, or PLT < 50 x 10^9 / L, it is up to the doctor to determine whether the drug can be used;

(11) Patients with active bleeding and peptic ulcer;

(12) Patients with other autoimmune diseases.

(13) Have used biological agents 6 months before enrollment;

(14) Added azathioprine, leflunomide, methotrexate, cyclosporine (or any other calcineurin inhibitors) within 1 month before enrollment, or adjusted the dosage of the above drugs within 1 month patient;

(15) Those who have participated in other drug clinical trials within 4 weeks.

4.3 Exit (loss)

Enrolled cases who did not complete the clinical protocol due to the following reasons should be considered dropped out:

(1) Some comorbidities, complications or special physiological changes have occurred in the subjects in the clinical trial, such as tumors, who are not suitable for continuing research;

(2) Poor compliance of subjects, affecting safety and efficacy evaluators;

(3) Subjects who have adverse events or serious adverse events, such as menopause for more than 3 months, hospitalization, severe infection or other serious adverse events, are not suitable for continuing the trial;

(4) Patients who are given prohibited drugs or treatments specified in the protocol after randomization;

(5) The subject is unwilling to continue the clinical trial and voluntarily withdraws;

(6) lost to follow-up;

(7) Other situations where the researcher judges that it is necessary to withdraw from the trial.

Processing principles:

For cases dropped out of the trial or lost to follow-up, researchers should actively take measures to complete the last test as much as possible for the analysis of its efficacy and safety.

For all dropped cases, the test conclusion form, and the reasons for the dropped

cases should be filled in the case report form. Generally, there are 6 types, that is, occurrence of adverse events (including adverse drug reactions and allergic reactions), lack of curative effect (disease or complications), violation of the trial protocol (including poor compliance), loss of follow-up (including subject voluntary withdrawal) test), and others.

4.4 Withdrawal

(1) After the case is enrolled, it is found that those who do not meet the inclusion criteria or meet the exclusion criteria;

(2) Those who have not taken medication after the case is enrolled;

(3) After the case is enrolled, it is automatically dropped out and lost to follow-up, and there is no post-treatment visit record.

The reasons for the excluded cases should be explained, and the case information should be kept complete for future reference. Statistical analysis of efficacy will not be performed, but those who have received at least one treatment and have safety records can participate in safety analysis as appropriate.

4.5 Termination

When clinical trial has not ended according to the plan, and the trial is stopped halfway. The purpose of the trial suspension is mainly to protect the rights and interests of the subjects, ensure the quality of the trial, and avoid unnecessary economic losses.

During the trial, if there are major mistakes in the established plan or major deviations in the operation process, making it difficult to evaluate the efficacy of drugs.

Treatment

5.1 Study drugs

（1） tofacitinib，5mg, tablet（patients pay）;

（2） prednison，5mg, tablet（patients pay）;

（3） Methylprednisolone，4mg tablet（patients pay）;

5.2 Oral administration

Treatment group: tofacitinib 5mg bid, 24 weeks of treatment

Positive control group: prednisone (or equivalent dose of methylprednisolone)

15mg/d, 2 weeks; 12.5mg/d, 2 weeks; 10mg/d, 6 weeks; 7.5mg/d, 6 weeks; 5mg/d, 8 weeks week, a total of 24 weeks.

If the disease relapses, the hormone dose is returned to the original dose.

(If tofacitinib treatment is ineffective for 6 weeks during the follow-up, the clinician can adjust the patient to the control group. If the pain is obvious, diclofenac 75mg qd or other similar drugs are allowed. Presence of osteoporosis: calcium carbonate D3 0.6g qd)

5.3 Randomization

Upon enrollment, after receipt of written informed consent and prior to any study-related procedures, each subject will be assigned a unique sequential random subject number for identification during the study, ranging from 001, 002, 003 and so on.

5.4 Treatment and follow up

Course of treatment: to observe the curative effect and safety of 12 weeks and 24 weeks.

Study procedures

6.1 Screening phase: (Weks -4 to Week 0) Screening the participants

(1) Sign the informed consent;

(2) Check the inclusion and exclusion criteria;

(3) Obtain past medical history and demographic data;

(4) Vital signs and physical examination;

(5) Disease assessment: PMR-AS score, pain VAS score, Patient self-assessment, Physician global assessment, morning stiffness time (min), difficulty in shoulder joint lifting (0, 1, 2, 3), difficulty in squatting and standing (0, 1, 2, 3)

(6) Laboratory examination:

Blood routine: red blood cell count, hemoglobin, white blood cell count, platelet count, neutrophil count, lymphocyte count

Urine routine: urine protein, urine red blood cells, urine white blood cells

Liver function: alanine aminotransferase (ALT), aspartate aminotransferase (AST), albumin (ALB)

Renal function: blood urea nitrogen (BUN), creatinine (CRE)

Blood glucose

Coagulation indicators: prothrombin time (PT), activated partial thromboplastin time (APTT), fibrinogen (FIB)

CRP, ESR

Immunological test: rheumatoid factor (RF), anti-CCP antibody (ACPA), Human leukocyte antigen B27 (HLA-B27), anti-nuclear antibody (ANA), anti-ENA antibody, anti-cardiolipin antibody, immunoglobulin and complement levels, anti-myeloperoxidase antibody (MPO), anti-proteinase 3 antibody (PR3).

(7) Electrocardiogram;

(8) Chest X-ray;

(9) Echocardiography;

(10) Abdominal B-ultrasound;

(11) Hepatitis B antigen, antibody, HBV-DNA; T-spot;

(12) PET/CT, MRI scan of shoulder and hip (optional);

(13) Bone mineral density;

(14) Record the concomitant medication and adverse events;

6.2 Follow-up 1 Weks 0

(1) Vital signs and physical examination;

(2) Randomization;

(3) Start drug treatment;

(4) Record concomitant medication and adverse events;

(5) Make an appointment for the next visit;

6.3 Follow-up 2 (Weks4±3 days), 3 (Weks8±3 days), 5 (Weks 16±3 days), 6 (Weks 20±3 days)

(1) Vital signs and physical examination;

(2) Disease assessment: PMR-AS score, pain VAS score, Patient self-assessment, Physician global assessment, morning stiffness time (min), difficulty in shoulder joint lifting (0, 1, 2, 3), difficulty in squatting and standing (0, 1, 2, 3)

(3) Laboratory examination:

Blood routine: red blood cell count, hemoglobin, white blood cell count, platelet count, neutrophil count, lymphocyte count

Urine routine: urine protein, urine red blood cells, urine white blood cells

Liver function: ALT, AST, ALB

Renal function: BUN, CRE

Blood glucose

CRP, ESR

(4) Drug treatment;

(5) Record the concomitant medication;

(6) Record adverse events;

(7) Make an appointment for the next visit;

6.4 Follow-up 4 (Weks 12±3 days), visit 7 (Weks 24±3 days)

(1) Vital signs detection and physical examination;

(2) Disease assessment: PMR-AS score, pain VAS score, Patient self-assessment, Physician global assessment, morning stiffness time (min), difficulty in shoulder joint lifting (0, 1, 2, 3), difficulty in squatting and standing (0, 1, 2, 3)

(3) Laboratory examination:

Blood routine: red blood cell count, hemoglobin, white blood cell count, platelet count, neutrophil count, lymphocyte count

Urine routine: urine protein, urine red blood cells, urine white blood cells

Liver function: ALT, AST, ALB

Renal function: BUN, CRE

Blood glucose

CRP, ESR

(4) drug treatment;

(5) Record the concomitant medication;

(6) Record adverse events;

(7) Make an appointment for the next visit;

Efficacy evaluations

7.1 The primary outcomes

The proportion of patients with PMR-AS<10 at 12 and 24 weeks of treatment.

7.2 The secondary outcomes

PMR-AS score, CRP, ESR at 12 and 24 weeks

7.3 Efficacy evaluations

PMR-AS<10：

(1) It indicated that the condition has improved significantly and the disease activity is low.

(2) Otherwise, it was thought as non-remission.

8. Safety

8.1 Adverse events

Record any unexpected or uncomfortable symptoms, signs, diseases, or the events which may lead to physical injury and was temporarily related to the drugs, but not necessarily causally related to the drug during the entire trial. It should be indicated that whether the adverse events were related to the investigational drug and whether they were related to polymyalgia rheumatica.

The possible adverse events include mild upper respiratory tract infection, mild urinary tract infection and herpes zoster. The incidence of the above symptoms is about 2%, and the corresponding treatment measures could be seen in the following part 10.12.

8.2 Lab test

During the study, laboratory tests were carried out as follows:

Blood routine: red blood cell count, hemoglobin, white blood cell count, platelet count, neutrophil count, lymphocyte count

Urine routine: urine protein, urine red blood cells, urine white blood cells

Liver function: ALT, AST, ALB

Renal function: BUN, CRE

Blood glucose

Coagulation indicators: PT, APTT, FIB

The normal value range of relevant laboratory test items should be obtained before the study begins. If the patient had obvious abnormal laboratory test results after taking the drug, he should be followed up to the normal range (or return to the baseline state). If it failed to return to normal (or baseline) within a certain period, the investigator should conduct etiological examination and diagnosis.

9. Concomitant diseases and treatment

9.1 Concomitant diseases

Concomitant diseases were the diseases present at the time when the informed consent was given, and would be recorded on the case report form.

9.2 Treatment

All other treatments administered at the time when the subjects entered the study or at any time during the study were considered as concomitant treatment, and the drugs used were recorded in the case report form in the form of generic names. The concomitant medication during the trial period must be necessary for the subjects and the dosage should be kept at the lowest level when the investigators thought that there is no interference with the trial drug.

When the drugs for concomitant diseases had to be used during the trial period due to certain diseases, dosage should be monitored in addition to complying with the above principles.

9.3 Prohibited drugs

Subjects who using prohibited drugs or treatments at any time during the study would be judged as failure and excluded from the study. The following drugs and treatments are prohibited at any time during the study:

（1) Other investigational drugs (biologic or nonbiologic). Investigational drugs include any drug that is not approved for sale in the country but is in use;

（2) Another clinical study of an experimental drug at the same time or a clinical

study of a non-drug treatment that might interfere with this study;

（3) Interleukin-6 (eg, Actemra),

（4) Other biologics (rituximab),

（5) Live vaccines are prohibited.

10. Handling of adverse event (AE) and serious adverse event (SAE)

It is the investigator's responsibility to discover and record events that meet the definitions of AE and SAE specified in the study protocol.

10.1 Definition of AE

An AE refers to any unfavorable medical event that occurs in a patient or clinical research subject using a certain drug, and it does not necessarily have a causal relationship with the treatment.

Therefore, an AE can be any unfavorable and unexpected sign (including abnormal laboratory test results of clinical significance), symptom or disease (new or exacerbation of pre-existing disease) temporarily associated with the drug; if marketed drugs should also include failure to produce the desired effect after use (that is, lack of effect), abuse and misuse.

Adverse reactions include:

（1）The test drug has good tolerance, and the incidence of adverse reactions is extremely low. If there is an allergic reaction, timely symptomatic treatment such as antihistamines or glucocorticoids can be given according to the general anti-allergic treatment method;

（2）Obvious or unforeseen aggravation or deterioration of the original disease;

（3）The exacerbation of the original chronic disease or the aggravation of the intermittent disease, manifested as an increase in frequency and/or intensity;

（4）The new disease is present or diagnosed after the use of the trial drug, even if it may have existed before the start of the study;

（5）Signs, symptoms or clinical sequelae arising from suspected interaction;

（6）Signs, symptoms or sequelae related to study drug overdose or other combined drug overdose (except for subject's own overdose);

（7） Reactions related to research drug allergy or toxicity;

（8） Obvious failure of expected pharmacological or biological response.

AE do not include:

（1）Medical or surgical examination and treatment process (such as endoscopy, appendectomy), but the disease that required these examinations is an AE;

（2）Circumstances where harmful medical events have not occurred (such as hospitalization for social accommodation or convenience of patients);

（3）Pre-existing diseases or conditions that existed or were discovered at the beginning of the study had expected periodic fluctuations, but did not worsen;

（4）The expected progression, symptoms, or signs associated with the disease or disorder, unless they are more severe than expected from the subject's disease.

10.2 Definition of SAE

A SAE is an AE that occurs at any dose level and meets any of the following conditions:

（1）cause death

（2）Threat to life

Note: Life-threatening means that the subject is in danger of death when the event occurs. This definition does not include events that could lead to death if the disease progresses further.

（3）Hospitalization or prolonged hospitalization

Note: In general, "inpatient" means that the subject is not suitable for observation or treatment in the outpatient or emergency department, but requires formal admission or emergency observation (usually at least overnight). Complications that occurred during hospitalization were considered AE. If the length of hospital stay was prolonged due to comorbidities or any other SAE criteria were met, it was also considered a SAE. Elective surgery performed because of a pre-existing condition that was not worse than baseline was not considered a SAE.

（4）Disability

Note: Disability refers to the substantial loss of a person's ability to live a normal life, excluding related minor illnesses such as simple headaches, nausea, vomiting, diarrhea, influenza, accidental injuries (such as ankle sprains), etc., although they

There may be some impact on activities of daily living, but not substantial (long-term) loss.

（5）Congenital malformations/abnormalities

（6）Certain situations that require medical or scientific judgment to decide whether to report urgently in this situation: although not resulting in death, immediate life-threatening or hospitalization but may cause harm to the subject, or may require medical or surgical treatment to prevent an important medical event that occurs as a result of one of the consequences listed above can be considered a SAE. Such medical events included invasive or malignant cancer, allergic bronchospasm requiring intensive care in the emergency room or at home, haematological dyscrasias or convulsions that did not result in hospitalization, and events of drug dependence or abuse.

10.3 Lack of efficacy

Lack of efficacy itself cannot be reported as an AE, and any symptoms/signs or sequelae caused by lack of efficacy need to be reported only if they meet the criteria for AE/SAE.

10.4 Abnormal clinical test results

Some abnormal laboratory results (such as clinical biochemistry, hematology, urinalysis) or other abnormal indicators (such as electrocardiogram, vital signs, etc.) are judged by the investigator to have clinical significance, if they meet the definition of AE in section 10.1 ("Definition of AE"), or the definition of a SAE in Section 10.2 ("Definition of SAE"), it must be recorded as an AE or as a SAE. Clinically significant abnormal laboratory test results and other abnormal findings that are found after treatment, or exist at the baseline assessment and aggravate after the start of the study, should be regarded as AE or SAE. However, clinically significant abnormal laboratory test results or other abnormal findings related to the disease under study are not included in AE or SAE unless the investigator determines that the subject's condition is more serious than expected. Abnormal laboratory results or other abnormal findings that existed or were detected but not exacerbated at the beginning of the study were also not included in AE and SAE.

It is up to the investigator to determine whether an abnormal laboratory test

result or other abnormal finding is clinically significant.

10.5 How to find AE

AE will be informed through oral questions and recorded on the CRF. Investigators or designees should use the same questions each time they ask about AE to avoid inter-subject variability. Researchers can ask:

"Have you felt any different since your last visit?"

In response to the subjects' answers to the above questions, the researcher can ask some related questions according to the patient's special complaints, such as:

How severe are the symptoms?

How often does it appear?

How long do general symptoms last?

Investigators also asked subjects about past unresolved AE. The investigator will assess the intensity, severity, relationship to the trial drug, and management of AE.

From before the start of the experiment to the end of the experiment or the corresponding exit visit, the researcher will ask the subjects the above questions.

10.6 Records of AE and SAE

When an AE/SAE occurs, the investigator is responsible for reviewing all relevant records (such as disease course, laboratory examinations, and diagnostic reports), and recording the information related to the event in the subject's case report form.

When recording adverse reactions, it should be indicated whether the adverse reactions are related to the test drug; and whether they are related to PMR. Researchers should try their best to judge the event based on symptoms, signs and/or other clinical data. In such cases, the diagnosis should be recorded as an AE and/or SAE rather than a subject's signs/symptoms.

10.7 Assessment of AE and SAE

10.7.1 Judgment of severity

During the study period, investigators should use their own clinical judgment to assess the severity of each AE and SAE reported. AE and SAE recorded in the case report form should be classified according to the following criteria:

Mild: events that only cause slight discomfort to the subject, do not affect daily

activities, and are relatively easily tolerated by the subject;

Moderate: Events that cause significant discomfort to the subject and interfere with normal daily activities;

Severe: Events leading to inability to perform normal daily activities.

Be careful not to confuse severe AE with SAE: Severity is a category used to measure the severity of events. Both AE and SAE can be rated as severe. SAE should be included.

10.7.2 Judgment of causality

Researchers must judge the relationship between each AE/SAE and the drug based on clinical experience. The relationship between the AE and the trial drug was evaluated according to five levels: definitely related to the drug, probably related, possibly related, probably irrelevant, and irrelevant.

（1）Definitely related: the time sequence of medication and reaction is reasonable; After withdrawal of the drug, reaction stops, or quickly alleviates or improves; the reaction reappears after repeated use; it is also supported by literature; and the influence of other confounding factors such as the original disease has been excluded;

（2）It is likely to be related: no history of repeated medication, the rest is the same as "definite" or although there is a combination of medication, the possibility of the reaction caused by the combination of medication can be basically ruled out;

（3）Possibly related: There is a close relationship between the medication and the time of the reaction, as evidenced by literature; however, there are more than one drug causing the adverse reaction, or the disease progression factors of the original disease cannot be excluded;

（4）Possibly irrelevant: the adverse reaction is not closely related to the time of medication, and the reaction performance does not match the known adverse reactions of the drug.

The development of the original disease may also have similar clinical manifestations;

（5）Irrelevance: There is no relationship between adverse reactions and medication time.

Adverse reactions refer to the sum of 1, 2, and 3.

Other causes, such as the natural history of the underlying disease, other

concomitant treatments, risk factors, and events temporally related to the trial drug, should be considered, and examined. Investigators should also consult the clinical investigator's brochure and/or the relevant information of the trial drug (if it is a commercially available drug) for evaluation.

Investigators may have little information when initially reporting a SAE, however, it is important for investigators to assess causality of each event before reporting escalation to the CRF for SAE. The researcher can change the judgment of causality according to the visit data and modify the corresponding CRF page accordingly. The judgment of causality is one of the necessary indicators.

The investigator should provide a causality assessment in the format of the SAE table in the CRF.

10.8 Visits for AE and SAE

Once an AE/SAE is reported, the investigator should visit each subject. All AE/SAE that have been recorded and persisted in the previous visit/re-visit should be re-examined in this visit/re-visit.

All AE/SAE must be followed up until their remission, stable condition, other explanations for the cause of the event, or loss of subjects. Once the issue is resolved, the AE/SAE section of the CRF should be updated in a timely manner. The investigator may add additional tests to the visit that may help clarify the nature and/or cause of the AE or SAE. This may include additional laboratory tests or studies, pathology tests, or consultation with other professionals.

New or updated information should be recorded on the initially completed CRF SAE page, and the investigator should sign and date the new or updated information.

10.9 Management and Reporting of SAE

Once the investigator finds that a SAE has occurred in the subject, he must take immediate action. The SAE report form should record all available event-related information as completely and in detail as possible. In the initial report, the investigator should evaluate the causality according to 10.7.2 (judgment of causality), and report to the ethics committee.

Reporting agency	Telephone	Fax	Primary contact

First Affiliated Hospital of Zhejiang University School of Medicine	**13906539996**	**0571-87235614**	Jin Lin

10.10 AE and SAE after the end of study

AE/SAE after the end of the study refer to any events that occur outside the visit period specified in 10.5. Researchers do not have to actively seek out AE and SAE that occurred in subjects who have completed clinical research and visits. However, if the investigator becomes aware of any SAE (including death) at any time after a subject has been withdrawn from the study, and the event is likely to be related to the study drug, the investigator should contact the subject and deal with it accordingly, make corresponding records, and report to the ethics committee.

10.11 AE and SAE related to trial

SAEs are related to participating in the study (such as operations, invasive examinations, and changes in existing treatment regimens), whether they occur before or after treatment, should be dealt with and recorded in time, and reported to the ethics committee.

10.12 Treatment for AE in this trial

1. Abnormal blood routine and liver function:

	Continue the original plan and give symptomatic treatment	Decrease dose of Tofacitinib and give symptomatic treatment	Stop Tofacitinib
Blood routine	WBC $\geq 3\times10^9$/L	3×10^9/L>WBC>2.5×10^9/L	WBC<2.5×10^9/L
Liver function	Transaminases elevated < 1.5-fold	1.5-fold ≤ Transaminases elevated < 3-fold	Transaminases elevated ≥ 3-fold

In the above situation, hepatoprotective drugs and agents for leucopenia can be added as appropriate. Researchers need to adjust the treatment regimen according to the correlation between adverse events and drugs. If the re-examination of liver function is normal, the original therapeutic dose can be restored; if abnormal liver

function occurs again, the dose should not be increased after the dose is reduced/stopped. The trial drug was discontinued for 8 consecutive weeks, and the patient withdrew from the study.

2. Drug allergy: withdraw from the study and treat according to clinical experience.

3. Mild upper respiratory tract infection, mild urinary tract infection, herpes zoster and other AE: They should be diagnosed and treated according to clinical diagnosis and treatment routines. It is controllable and treatable.

If SAE occur, they should be withdrawn from the trial.

11. Drugs adherence

After the trial, we will evaluate the adherence of the patient, record the doses of drugs used, and record whether the patient uses the trial drug.

12. Withdrawal

12.1 Subject completes the trial

Subjects are considered to complete the trial when they have completed all elements specified in each scheduled visit in accordance with the requirements of the trial.

12.2 Subject withdraws from trial

Any subject who entered the trial (signed informed consent) failed to complete the experiment in accordance with the requirements of the above definition for any reason, and would be considered an withdraw from trial, regardless of whether they used the trial drug.

94 patients are expected to be selected randomly, and the subjects participating in the trial will get the unique random number.

Each subject can withdraw from the study at any stage, any reasons (special or non -special) without being discriminated against. The treatment of patients will not be restricted. Under appropriate cases, the researchers will use conventional treatment methods to treat patients.

Patients who withdraw should be visited as much as possible. Patients who

withdraw after randomization should complete the current visit if they withdraw at the time of the scheduled visit. All subjects who withdraw from the trial after randomization should complete all the evaluations required by the visit 3.

The case report forms of all the subjects participating in the randomization must be filled in completely, and the reasons for all withdraw subjects who were randomized but did not complete the trial should be recorded on the "trial summary" of the case report form. In addition, the date of last dose for subjects who withdrew from the trial must be recorded on the "trial completion summary".

12.3 Screening and baseline assessment failure

Patients withdraw from the trial prior to randomization are considered screening failures. The reason for withdrawing from the trial should be recorded in the patient screening entry form.

13. Data management

（1）Researchers should carry data based on the subject's original observation records in time, completely, correctly, and clearly into the case report form.

（2）Confirm that the report forms of all cases are filled in correct and completely, and consistent with the original information. If there are errors and omissions, the researchers are required to correct them in time. When modified, the original record must be clearly visible, and the correction must be signed by the researcher and indicated the date.

（3）There should be a special record for the transmission of the complete case report form between researchers and data manager. When a record is received, there should be a signature, and the record needs to be properly maintained.

（4）Data manager should check again before the data entry, promptly notify the researcher of any problems and request answers from the researcher. The exchanges of questions and answers between researchers should be used in the form of question tables, and the question table should be preserved properly.

（5）Before data entry, date mangers must understand the content and coding of each item of the observation form, and record the encoding work process to the coding book for preservation. Database naming should be standardized, easy to read, and easy to find. And ensure that it is correct, safe and confidential.

（6）The data entry clerk enters data using secondary entry. Problems or unexpected

situations found in the data entry process should be registered and reported in time so that the problems can be dealt with quickly. After the data entry is finished, some observation forms should be sampled to understand the quality of the entry and analyze and deal with any problems.

（7）Data mangers shall, together with the main researchers, formulate data range inspection and logical inspection content according to the scope of each indicator value and relationship in the report form in the case. And write the corresponding computer program, control the error data input before the input, and find out the reason for the error to correct it. All error content and modification results should be recorded and preserved properly.

（8）The original case report form, after completing data entry and verification as required, is archived and kept in a numbered order with a searchable table of contents, etc., filled in for reference. Electronic data files include databases, inspection procedures, analysis procedures, analysis results, encoding books, and explanation files. They should be categorized and stored, and multiple backups are stored on different disks or recording medias to properly store and prevent from damage. All original archives should be stored in accordance with the prescribed period of Chinese "Good Clinical Practice" .

14. Statistical analysis

Finalize according to data characteristics. This plan provides statistical conventional requirements.

14.1 Sample size determination

According to perspective of effectiveness and the statistical requirements, the differential test is adopted, and the two-sided test is used $\alpha=0.05$, $\beta = 0.10$ (90% confidence). The CR rate at 12 and 24 weeks is the primary endpoint, and the CR rate of the patients in the experimental group is predicted. The rate was 95%, the CR rate of the control group was 85%, the margin of superiority/non-inferiority was 0.1, the sample distribution ratio of the two groups was 1:1, calculated by PASS software, 38 cases are needed in each group. According to the 18-20% dropout, finally a minimum of 47 cases per group is required. The minimum total sample size was 94 cases, with 47 cases in the test group and 47 cases in the control group.

14.2 Analysis set

（1）Per-Protocol Set (PPS)：This refers to the collection of cases that met the inclusion criteria, did not meet the exclusion criteria, and completed the treatment protocol, i.e., Cases that met the trial protocol, had good compliance, and completed the required completion of the CRF were analyzed (PP analysis). PP analysis was mainly used for the main efficacy indicators.

（2）Safety Set (SS)：At least one treatment is received, and actual data recorded by security indicators. Safety missing values were not allowed to be carried forward. We include partially excluded cases for evaluation, such as those older than the inclusion criteria, but not those in which the use of prohibited drugs rendered a safety determination impossible. The incidence of adverse reactions was determined using the number of cases in the safety set as the denominator.

14.3 Statistical Methods

14.3.1 Patient enrollment analysis

- List the overall selection and complete cases, and determine the two analysis data sets (PPS, SS).
- List the cases and reasons for falling-off and exclusion.

14.3.2 Population data and baseline analysis

Descriptive statistics demographic information and other baseline characteristic values：

- Continuous variables calculate the cases, mean, standard deviation, median, minimum, and maximum values.
- Calculation and grade data count frequency and constituent ratio.
- Inference statistical results (*P* value) are listed as descriptive results.

14.3.3 Efficacy analysis

Efficacy analysis

- The comparison of the proportion of patients with PMR-AS<10 at 12 and 24 weeks of treatment was analyzed between two groups by Fisher's exact test.
- After measuring values of PMR-AS scores, CRP, and ESR at 12 and 24 weeks of treatment, we compare differences between two groups at 12 and 24 weeks using repeated ANOVA.

14.4 Safety analyses

- Calculate the incidence of adverse events and adverse reactions;
- Calculate the incidence of serious adverse events;
- Frequency and relative frequency of adverse events and reactions are in the subsystem and percentages are calculated, then analyzed by Fisher exact test;
- List the detailed list of adverse events;
- List the detailed list of adverse reactions
- Laboratory inspections, electrocardiogram, and physical examinations of "normal transition" or "abnormal intensification" after trial;
- List laboratory examination, electrocardiogram, physical examination abnormal cases and clinical interpretations
-

14.5 Statistical software

- Analysis using SPSS 18 software
- All statistical tests were performed using bilateral test, and a p-value less than or equal to 0.05 would be considered statistically significant for the difference tested
- The detailed statistical method will be provided in the statistical analysis plan.

14.6 Interim analysis

There will be no interim analysis for this study. If special circumstances occur in the trial, such as inaccurate efficacy or safety issues, they will be discussed by researchers and staffs together.

15. Research management

15.1 Comply with GCP requirements

This trial shall be performed in accordance with the requirements of the Chinese Good Clinical Practice (GCP).

15.2 Privacy of subjects

When filling in and managing the report form, the privacy of the patient should be considered, such as the patient's random number to represent the patient, etc. The patient's initials may also be used as a surrogate for his or her name

15.3 Informed consent

Prior to starting this trial, we must explain the content in subject's informed consent to each participant that can easily understand. Then we obtain written informed consent from the patient to voluntarily participate in this trial. Enter the date written informed consent was obtained in the CRF.

（1）Nature and purpose of the research;

（2）Research process

（3）Benefit and risk

（4）Other alternative treatment

（5）The right to subject: Patients refuse to participate in this trial at any time, they can withdraw from this trial, and the patient's rights will not be affected by any way.

（6）Confidentiality agreement

（7）What the patient must follow

（8）And any matters believed to protect the rights of the patient

15.4 Protocol revision procedure

When the plan must be modified or suspended in this trial, the principal researchers should immediately notify all personnel participating in the trial. In the case of major modifications, new ethics committee approval should be obtained before implementing.

15.5 Case report form recording and modification procedures

True and accurate recording of trial data.

If you need to make any modifications, please keep the record of modification legible, and sign the date. If the scope of the amendment is large or the changes are significant, the modified person should indicate the reason for the revision and modification, and sign it at the same time.

15.6 Quality Control

（1）Adopting standard operating procedures ensure the quality control of study and quality assurance systems for this clinical trial.

（2）All observed results and abnormalities findings in clinical trials should be carefully verified and recorded in time to ensure the reliability of data. Various

instruments, equipment, reagents, standard products, etc. used in various inspection items in clinical trials should have strict quality standards and ensure that they work in normal state. The records and metastasis of clinical data must be responsible for experienced physicians, and special persons supervise or verify to ensure the scientific and accuracy of the data. Various conclusions of clinical trials must be derived from the original data.

（3）Doctors responsible for the test should fill in the case report form (CRF) in a complete, detailed, accurate, and timely manner. After the signature of the hand -to senior physician is confirmed, it is submitted or saved in accordance with the prescribed procedures. All data related to the trial should be centralized and analyzed.

（4）Establish procedures for data storage, data transmission, and data query. The custody information includes: the subject's research medical records, imaging materials, CRF, pharmaceutical registration form, subject screening form, subject recognition code table, serious adverse event report forms, GCP forms required to fill in each hospital, visit report form and various original medical documents. The passed data includes: the subject random table, CRF, serious adverse event report forms and data and information to be used in summarizing information.

（5）When we sum up and analyze the results of clinical trials, standardized statistical analysis methods must be adopted, and those who are familiar with biological statistics must be invited to participate.

15.7 Data storage

（1）The original data is kept by the research institution.

（2）Retention period until 5 years after the end of the study.

（3）All information of this clinical trial belongs to the first hospital affiliated to Zhejiang University school of medicine.

15.8 Schedule

Because of the low prevalence of the disease, the duration of the study was set at 2 years in order to collect the full number of patients

16. Paper publication

The researchers and other staff of the research center shall keep confidential all information provided by the First Affiliated Hospital of Zhejiang University School of Medicine and all data generated by the center of the research during the course of the study (except medical records of the subjects). Researchers or other staff of the research center may not use the information, data, or records for any purpose other than for the purpose of this study. These restrictions do not apply to information that: (1) has been made publicly available through no error on the part of the investigator or staff; (2) must be disclosed for the purpose of gaining the confidence of an academic or ethics committee to evaluate the study; or (3) must be disclosed in order to provide appropriate health care to the subjects participating in the study.

17. Reference

[1] 国家食品药品监督管理局. 药物临床试验质量管理规范（GCP2003 版）.

[2] 新药（西药）临床研究指导原则.卫生部药政局（1993 版）.

Table 1 1982 Chuang Criteria for PMR

1	age＞ 50 years
2	>1 month bilateral aching and stiffness of at least two of the following areas: Neck or torso, shoulders or proximal arms, hips or proximal thighs; exclusion of other causes
3	ESR>40mm/h
4	Exclude diseases other than giant cell arteritis

All criteria must be met

Table 2 2012 EULAR/ACR Classification Criteria for PMR

Required Criteria:	age＞ 50 years，bilateral shoulder aching, abnormal ESR/CRP

	Points Without Ultrasonography （0-6）	Points With Ultrasonography （0-8）
Morning stiffness duration＞ 45 min	2	2
Hip pain or limited range of movement	1	1
Absence of RF or ACPA	2	2
Absence of other joint involvement	1	1
≥1 shoulder with subdeltoid bursitis and/or biceps tenosynovitis and/or glenohumeral synovitis (either posterior or axillary) and ≥1 hip with synovitis and/or trochanteric bursitis	/	1
Both shoulders with subdeltoid bursitis, biceps tenosynovitis, or glenohumeral synovitis	/	1

A score ≥4 without ultrasonography is categorized as PMR, and a score ≥5 with ultrasonographic finding is categorized as PMR.

Table 3 Polymyalgia rheumatica disease activity (PMR-AS) score

CRP (mg/dl)	
Patient self-evaluation (0-10 visual scale)<br>0=no pain, 10=no pain tolerable	
Physician global assessment (0-10 Visual Scale)<br>0=no pain, 10=no pain tolerable	
Morning stiffness time min x0.1	
Shoulder joints（0-3）<br>0=lifted above the shoulder girdle, 1=to the shoulder girdle,	

2=below the shoulder girdle, 3=cannot lift	
Total	

A score of less than 10 indicates disease remission or low disease activity

Difficulty squatting and standing due to hip muscle involvement, score,
0 = no difficulty, 1 = can squat and stand with the help of others or upper body strength, 2 = can squat with the help of others or upper body strength, but cannot stand, 3 = cannot squat and stand.

托法替布对风湿性多肌痛的治疗疗效和安全性前瞻性研究

临床研究负责单位：浙江大学医学院附属第一医院

方案制订日期：**2020** 年 **7** 月 **19** 日

主要研究者：　林进

版本日期：**2020** 年 **7** 月 **19** 日

版本号：**2.0**

方案修订历史

版本	日期	版本说明
1.0	2020-06-17	初稿
2.0	2020-7-19	修订稿

本临床试验研究方案属机密资料，为浙江大学医学院附属第一医院所有。

研究者声明及签字页

该项研究为前瞻性临床研究，研究中采用的方法学、药物均已被临床广泛应用。本研究将设立严格的不良事件监测制度，仔细记录所有出现的不良事件并做到及时有效处理。如发生任何严重不良事件或重要的不良事件，无论是否与研究干预有关，也无论是否已实施干预操作，均必须及时通知研究发起人，并根据情况及时决定是否停止研究。研究者保证对受试者的个人资料做到严格保密：所有受试者信息及影像将以编号而非姓名加以标识；可以识别身份的信息将不会透露给研究小组以外的成员，除非获得受试者许可；所有的研究成员和研究发起人都被要求遵循保密原则；所有研究档案将保存在有锁的档案柜中，仅供研究人员查阅；研究实施过程中和完成后，允许政府管理部门或伦理委员会成员按规定对受试者的个人资料进行抽查监督；这项研究结果发表时，将不会披露个人的任何资料。

研究者签名

我已经阅读和同意此文件提出的方案，并对方案相关内容予以认同，在试验过程中将会严格遵守中华人民共和国法律法规及相关规章制度，履行研究者的职责并遵守保密规定。

研究单位：________________________

研究者姓名：______________________

研究者职务：______________________

研究者签字：______________________

签字日期：________________________

目录

摘　要 **5**
缩略语表 **7**
临床研究流程图 **8**
1. 前言 **10**
2. 研究目的 **10**
3. 研究设计 **10**
3.1 总体设计 10
3.2 随机化分组 10
3.3 样本量与分配 10
4. 研究人群 **11**
4.1 入选标准 11
4.2 排除标准 11
4.3 退出（脱落）标准 12
4.4 剔除标准 12
4.5 终止标准 12
5. 治疗 **13**
5.1 试验药物 13
5.2 用药方法 13
5.3 随机化过程 13
5.4 疗程 13
6. 研究过程 **14**
6.1 筛选期:（第-5 周～0 周）患者入选 14
6.2 访视 1 第 0 周 15
6.3 访视 2（第 4 周±3 天）、访视 3（第 8 周±3 天）、访视 5（第 16 周±3 天）、访视 6（第 20 周±3 天） 15
6.4 访视 4（第 12 周±3 天）、访视 7（第 24 周±3 天） 15
7. 疗效评价 **16**
7.1 主要疗效指标 16
7.2 次要疗效指标 16
7.3 疗效评定标准 16
8. 8. 安全性评价 **16**
8.1 不良事件 16
8.2 实验室检查 17
9. 伴随疾病和治疗 **17**
9.1 伴随疾病 17
9.2 伴随治疗 17
9.3 禁用药物 17
10. 不良事件及严重不良事件 **18**
10.1 不良事件的定义 18
10.2 严重不良事件的定义 19
10.3 缺乏疗效 20
10.4 临床检验结果异常和其它异常指标作为不良事件或严重不良事件 20

10.5 发现不良事件的方法，频率和时限 20
10.6 不良事件和严重不良事件的记录 21
10.7 不良事件和严重不良事件的评估 21
10.8 不良事件和严重不良事件的访视 22
10.9 严重不良事件的处理和报告 22
10.10　研究结束后的不良事件和严重不良事件 23
10.11 与参加试验有关的不良事件和严重不良事件 23
10.12 本研究不良反应及处理方法 23
11.　用药依从性 24
12.　退出研究 24
12.1 受试者完成试验 24
12.2 受试者退出试验 24
12.3 筛选和基线评估失败 24
13.　数据管理 24
14.　统计分析 25
14.1 样本量估算 25
14.2 分析数据集 25
14.3 统计方法与内容 26
14.4 安全性分析 26
14.5 统计软件与一般要求 27
14.6 期中分析 27
15.　研究管理 27
15.1 遵从 GCP 的要求 27
15.2 保护受试者的隐私权 27
15.3 知情同意书 27
15.4 方案的修订程序 28
15.5 病例报告表记录及修改程序 28
15.6 质量控制和质量保证 28
15.7 资料保存 28
15.8 项目进度计划 29
16.　论文发表 29
17.　参考文献 29
附表一　**1982 年风湿性多肌痛分类标准 30**
附表二　**2012 年风湿性多肌痛分类标准 30**
附表三　风湿性多肌痛疾病活动度**(PMR-AS)**评分 **.......... 30**

摘　要

方案名称	托法替布对风湿性多肌痛的治疗疗效和安全性前瞻性研究
适应症	风湿性多肌痛患者
试验目的	观察托法替布治疗风湿性多肌痛的有效性及安全性
研究单位	浙江大学医学院附属第一医院
试验人群	符合入选标准且不在排除标准中的患者
试验样本量	本次临床试验的托法替布治疗组 47 例，对照治疗组 47 例。
试验用药物	1. 托法替布，规格：5mg 片剂（患者自付）;<br>2. 泼尼松，规格：5mg 片剂（患者自付）;<br>3. 甲泼尼龙，规格：4mg 片剂（患者自付）;
给药方案	**试验组：**托法替布 5mg bid，疗程24周<br><br>**对照组：**泼尼松（或等效剂量甲泼尼龙） 15mg/d，2 周; 12.5mg/d，2 周；10mg/d，6 周；7.5mg/d，6 周；5mg/d，8 周，共 24 周。<br>如果病情复发，激素剂量恢复到原来的剂量。<br><br>主要终点：12、24周PMR-AS <10的患者比例<br>次要终点：12、24周访视时PMR-AS评分，CRP，ESR<br><br>(若随访过程中托法替布治疗6周无效，临床医生可以调整该患者到对照组。疼痛明显，允许使双氯芬酸 75mg qd或其他同类药品<br>存在骨质疏松：碳酸钙 D3 0.6g qd)
入选标准	1. 符合 1982 年标准（附表 1）或 2012 年 ACR/EULAR 风湿性多肌痛诊断标准（附表 2）；未使用过激素和免疫抑制剂 3 个月以上；<br>2. 高度活动风湿性多肌痛的定义：疾病活动度评分 PMR-AS (附表 3)>10 分；<br>3. 年龄 50-75 岁，体重 45-80Kg；<br>4. ESR>40mm/h 或者 CRP >100mg/L（10mg/dl）;<br>5. 签署知情同意书。
排除标准	1. 已知对托法替尼、强的松或甲强的松过敏的患者;<br>2. 患有巨细胞动脉炎、系统性红斑狼疮、类风湿关节炎等风湿性疾病的患者;<br>3.严重骨关节炎患者;<br>4. 患有严重急性、慢性或复发性感染(如肺炎或肾盂肾炎、复发性肺炎、慢性支气管扩张、肺结核等)的受试者;<br>5. 肝功能异常患者(ALT/AST 升高 2 倍);<br>6. 孕妇或哺乳期妇女;

	7. 有恶性肿瘤史且不符合以下条件的患者(恶性肿瘤患者在筛查前已成功治疗 5 年以上且无复发证据);<br>8. 既往有视野障碍或仅单眼有功能障碍者、白内障患者；<br>9. 代偿失调的心功能不全或严重高血压或糖尿病患者，即收缩压高于 160mmHg，或舒张压高于 100mmHg；<br>10. 血常规如有异常，WBC < $2x10^9$/L，或 HGB < 60g/L，或 PLT < $50x10^9$/L，由医生判断是否可以用药；<br>11. 活动性出血和消化道溃疡者；<br>12. 合并其他自身免疫性疾病者；<br>13. 入组前 6 个月使用过生物制剂；<br>14. 入组前 1 个月内新加用硫唑嘌呤、来氟米特、甲氨蝶呤、环孢素（或任何其他钙神经素抑制剂），或 1 个月内调整上述药物剂量的患者；<br>15. 4 周内参加过其他药物临床试验者。
随访时间点	筛查、第 0、4、8、12、16、20、24 周共 8 次。
疗效指标	**主要疗效指标：**<br>12、24 周 PMR-AS <10 的患者比例；<br>**次要疗效指标：**<br>PMR-AS评分，CRP，ESR
安全性指标	1. 观察不良事件和不良反应发生情况如感染、带状疱疹，皮疹，腹泻，大便 OB 阳性；<br>2. 血、尿常规化验，肝肾功能等实验室异常发现；<br>3. 一般体格检查（如心率和血压）异常发现；<br>4. 骨密度，腰椎骨折，股骨头坏死；<br>5. 青光眼等；
统计方法	主要疗效指标采用 PPS 分析集；<br>次要疗效指标采用 PPS 分析集；<br>安全性指标采用 SS 分析集。

缩略语表

英文缩写	全称
AE	不良事件
ALT	丙氨酸氨基转移酶
ANA	抗核抗体
APTT	部分活化凝血酶原时间
AST	天门冬氨酸氨基转移酶
BUN	尿素氮
CR	完全缓解
CRE	肌酐
CRF	病例报告表
CRP	C 反应蛋白
ECG	心电图
ESR	血沉
GCP	药物临床试验质量管理规范
HGB	血红蛋白
MMF	吗替麦考酚酯
NR	无反应
PPS	符合方案数据集
PR	部分缓解
RBC	红细胞计数
SAE	严重不良事件
SS	安全性数据集
WBC	白细胞计数

临床研究流程图

随访周数	第-4 周<br>～0 周	访视 1<br>第 0 周	访视 2<br>第 4 周<br>±3 天	访视 3<br>第 8 周<br>±3 天	访视 4<br>第 12 周<br>±3 天	访视 5<br>第 16 周<br>±3 天	访视 6<br>第 20 周<br>±3 天	访视 7<br>第 24 周<br>±3 天
签署知情同意书	√							
入选/排除标准	√							
人口统计学资料	√							
既往病史	√							
生命体征	√	√	√	√	√	√	√	√
体重、身高	√							
体格检查	√	√	√	√	√	√	√	√
疾病活动度指标：PMR-AS 评分	√		√	√	√	√	√	√
患者疼痛 VAS 评分	√		√	√	√	√	√	√
医生总体评价	√		√	√	√	√	√	√
晨僵时间(min)	√		√	√	√	√	√	√
上臂抬起困难 0,1,2,3	√		√	√	√	√	√	√
下蹲站起困难 0,1,2,3	√		√	√	√	√	√	√
血沉	√		√	√	√	√	√	√
C-反应蛋白	√		√	√	√	√	√	√

血常规[1]	√		√	√	√	√	√	√
尿常规[2]	√		√	√	√	√	√	√
肝功能[3]	√		√	√	√	√	√	√
肾功能[4]	√		√	√	√	√	√	√
血糖	√		√	√	√	√	√	√
凝血指标[5]	√							√
免疫学检查[6]	√							
乙肝抗原和抗体，HBV-DNA	√							
T-spot	√							
胸片	√							
PET 检测(非必需)	可选							可选
肩和臀部MRI(非必需)	可选							可选
骨密度	√							可选
心电图	√							
超声心动图	√							
随机		√						
合并用药	√	√	√	√	√	√	√	√
不良事件	√	√	√	√	√	√	√	√

注：1. 血常规：红细胞计数、血红蛋白、白细胞计数、血小板计数、中性粒细胞绝对值、淋巴细胞绝对值；

2. 尿常规：尿蛋白、尿红细胞、尿白细胞；

3. 肝功能：丙氨酸氨基转移酶（ALT）、天门冬氨酸氨基转移酶（AST）、白蛋白（ALB）；

4. 肾功能：尿素氮（BUN）、肌酐（CRE）；

5. 凝血指标：凝血酶原时间（PT）、活化部分凝血活酶时间（APTT）、纤维蛋白原（FIB）；

6. 免疫学检查：RF、抗CCP抗体、HLA-B27、抗核抗体、抗ENA抗体、抗心磷脂抗体、免疫球蛋白和补体水平、MPO、PR3

前言

风湿性多肌痛（PMR）在 50 岁以上的老年患者多发，表现为颈部、肩关节、肩胛带肌和骨盆带肌肉疼，是一种血管炎症，严重影响患者的生活质量。目前糖皮质激素是 PMR 的主要治疗药物，可以迅速缓解疼痛，下降炎症指标，改善生活质量，但总体激素治疗时间至少 1 年以上，激素减量时容易复发；这个患者群本身也存在很高比例的骨质疏松，糖尿病，心血管疾病等慢性疾病，同时糖皮质激素可导致多种副作用，如骨质疏松，消化性溃疡，类固醇糖尿病，青光眼，继发感染，代谢紊乱等，因此，寻找更好的治疗药物是临床迫切需要解决的问题。托法替布已被批准用于治疗高炎症性疾病如类风湿关节炎，快速缓解关节症状，减轻炎症反应。已经有个案报道（https://www.isr.ie/abstracts/a-review-of-patients-started-on-janus-kinase-inhibitor-jaki-in-university-hospital-waterford/）托法替布治疗 3 例 PMR 患者，有效改善 PMR 患者的症状，但目前缺乏系统的临床研究证实该药物对 PMR 的临床疗效。本课题旨在明确托法替布对 PMR 的安全性和有效性。为了观察托法替布治疗 PMR 的有效性和安全性，现拟进行本临床研究。

研究目的

本课题旨在探讨托法替布治疗PMR的有效性和安全性。

研究设计

3.1 总体设计

本研究遵循GCP原则，开展随机、单中心、前瞻性研究的设计方法。整个试验将按本方案由本研究中心完成。

3.2 随机化分组

随机化的实施应用信封法产生随机数字的方法进行随机化分组。

3.3 样本量与分配

3.3.1 样本量

本次临床试验的托法替布治疗组 47 例，传统治疗组 47 例。

3.3.2 病例分配

将符合入选标准，且不在排除标准中的病例作为受试者。

研究人群

4.1 入选标准

（1） 符合 1982 年标准（附表 1）或 2012 年 ACR/EULAR 风湿性多肌痛诊断标准（附表 2）；患者入组前 3 个月未使用过激素和免疫抑制剂治疗；

（2） 高度活动风湿性多肌痛的定义：疾病活动度评分 PMR-AS (附表 3)>10 分；

（3） 年龄 50-75 岁，体重 45-80Kg；

（4） ESR>40mm/h 或者 CRP >100mg/L（10mg/dl）；

（5） 签署知情同意书。

4.2 排除标准

（1） 已知托法替布、泼尼松、甲泼尼龙过敏者；

（2） 巨细胞动脉炎，系统性红斑狼疮、类风湿关节炎等风湿性疾病；

（3） 严重骨性关节炎患者；

（4） 患有任何严重的急性、慢性或复发性感染的受试者（例如肺炎或肾盂肾炎活动期、复发性肺炎、慢性支气管扩张症、结核病等）；

（5） 肝功能异常（谷丙或谷草转氨酶高于正常值上限 2 倍）；

（6） 妊娠或哺乳期女性；

（7） 有恶性肿瘤史且不符合以下情况（患基底细胞癌且已得到适当治疗；患其他恶性肿瘤但在筛选前已成功治疗超过 5 年且没有任何复发证据者）；

（8） 既往有视野障碍或仅单眼有功能障碍者、白内障患者；

（9） 代偿失调的心功能不全或严重高血压或糖尿病患者，即收缩压高于 160mmHg，或舒张压高于 100mmHg；

（10） 血常规如有异常，WBC < $2x10^9$/L，或 HGB < 60g/L，或 PLT < $50x10^9$/L，由医生判断是否可以用药；

（11） 活动性出血和消化道溃疡者；

（12） 合并其他自身免疫性疾病者；

（13） 入组前 6 个月使用过生物制剂；

（14） 入组前 1 个月内新加用硫唑嘌呤、来氟米特、甲氨蝶呤、环孢素（或任何其他钙神经素抑制剂），或 1 个月内调整上述药物剂量的患者；

（15） 4 周内参加过其他药物临床试验者。

4.3 退出（脱落）标准

因以下原因未完成临床方案的入组病例应视为脱落：

（1）临床试验中受试者发生了某些合并症、并发症或特殊生理变化，不适宜继续进行研究者，如肿瘤；

（2）受试者依从性差，影响安全性和疗效评价者；

（3）发生不良事件或严重不良事件，如停经大于 3 个月、住院、严重感染或其他严重不良事件，不适宜继续接受试验的受试者；

（4）在随机之后加用方案规定的禁用药物或治疗的患者；

（5）受试者不愿意继续进行临床试验，主动提出退出者；

（6）失访；

（7）研究者判断需要退出试验的其他情况。

处理原则：

对于中途退出试验病例或失访脱落病例，研究者应积极采取措施，尽可能完成最后一次检测，以备对其疗效和安全性进行分析。

所有脱落病例，均应在病例报告表中，填写试验结论表及病例脱落的原因。一般情况下有 6 种，即发生不良事件（包括药品不良反应和过敏反应）、缺乏疗效（病情恶化或出现并发症）、违背试验方案（包括依从性差）、失访（包括受试者自行退出试验）、和其他。

4.4 剔除标准

（1）病例入选后，发现不符合纳入标准或符合排除病例标准者；

（2）病例入选后未曾用药者；

（3）病例入选后即自动脱落失访，无治疗后访视记录者。

剔除的病例应说明原因，病例资料应保存完整备查。不作疗效统计分析，但至少接受一次治疗，且有安全性记录者，视情况可参加安全性分析。

4.5 终止标准

指临床试验尚未按方案结束，中途停止全部试验。试验中止的目的主要是为了保护受试者权益，保障试验质量，避免不必要的经济损失。

试验过程中发现所定试验方案有重大失误或操作过程中发现重大偏差，难以对药物进行评价；

治疗

5.1 试验药物

（1）　托法替布，规格：5mg 片剂（患者自付）；

（2）　泼尼松，规格：5mg 片剂（患者自付）；

（3）　甲泼尼龙，规格：4mg 片剂（患者自付）；

5.2 用药方法

试验组：托法替布 5mg bid，疗程24周

对照组：泼尼松（或等效剂量甲泼尼龙） 15mg/d，2 周; 12.5mg/d，2 周；10mg/d，6 周；7.5mg/d，6 周；5mg/d，8 周，共 24 周。

如果病情复发，激素剂量恢复到原来的剂量。

主要终点：12、24周PMR-AS <10的患者比例

次要终点：2、4、8、12、16、20、24周访视时PMR-AS评分，CRP，ESR，疼痛VAS评分，乏力VAS评分，患者总体评价，医生总体评价，晨僵时间（min），肩关节抬起程度（0、1、2、3）；下蹲和站立（0、1、2、3）

(若随访过程中托法替布治疗6周无效，临床医生可以调整该患者到对照组。疼痛明显时，允许使双氯芬酸 75mg qd或其他同类药品。存在骨质疏松时：碳酸钙D3 0.6g qd)

5.3 随机化过程

入选时，在收到书面知情同意书之后和进行任何研究相关程序之前，将为每名受试者分配一个专有的顺序性受试者编号，以用于研究期间进行识别，该编号从001、002、003开始依次类推。

5.4 疗程

疗程：观察 12 周、24 周的疗效和安全性。

研究过程

6.1 筛选期：（第-5 周～0 周）患者入选

（1）签署知情同意书；

（2）核查入选、排除标准；

（3）取得病史和人口学资料；

（4）生命体征检测和体格检查；

（5）PMR-AS评分、疼痛VAS评分、患者总体评价、医生总体评价、晨僵时间（min）；肩关节抬起程度（0、1、2、3）；下蹲和站立（0、1、2、3）

（6）实验室检查：

血常规：红细胞计数、血红蛋白、白细胞计数、血小板计数、中性粒细胞绝对值、淋巴细胞绝对值

尿常规：尿蛋白、尿红细胞、尿白细胞

肝功能：丙氨酸氨基转移酶（ALT）、天门冬氨酸氨基转移酶（AST）、白蛋白（ALB）

肾功能：尿素氮（BUN）、肌酐（CRE）

血糖

凝血指标：凝血酶原时间（PT）、活化部分凝血活酶时间（APTT）、纤维蛋白原（FIB）

CRP、ESR

免疫学检查：RF、抗CCP抗体、HLA-B27、抗核抗体、抗ENA抗体、抗心磷脂抗体、免疫球蛋白和补体水平、MPO、PR3

（7）心电图；

（8）胸部X线；

（9）超声心动图；

（10）腹部B超；

（11）乙肝抗原、抗体、HBV-DNA；T-spot；

（12）PET/CT，肩和臀部MRI（非必需）；

（13）骨密度；

（14）记录合并用药及不良事件；

6.2 访视 1 第 0 周

（1）生命体征检测和体格检查；

（2）随机；

（3）开始药物治疗；

（4）记录合并用药及不良事件；

（5）预约下次访视时间；

6.3 访视 2（第 4 周±3 天）、访视 3（第 8 周±3 天）、访视 5（第 16 周±3 天）、访视 6（第 20 周±3 天）

（1）生命体征和体格检查；

（2）疗效评价：PMR-AS 评分、疼痛 VAS 评分，患者总体评价，医生总体评价，晨僵时间（min），肩关节抬起程度（0、1、2、3）；下蹲和站立（0、1、2、3）

（3）实验室检查：

血常规：红细胞计数、血红蛋白、白细胞计数、血小板计数、中性粒细胞绝对值、淋巴细胞绝对值

尿常规：尿蛋白、尿红细胞、尿白细胞

肝功能：丙氨酸氨基转移酶（ALT）、天门冬氨酸氨基转移酶（AST）、白蛋白（ALB）

肾功能：尿素氮（BUN）、肌酐（CRE）

血糖

ESR

CRP

（4）药物治疗；

（5）记录合并用药；

（6）记录不良事件；

（7）预约下次访视时间；

6.4 访视 4（第 12 周±3 天）、访视 7（第 24 周±3 天）

（1）　生命体征和体格检查；

（2）　疗效评价：PMR-AS 评分、疼痛 VAS 评分，患者总体评价，医生总体评价，晨僵时间（min），肩关节抬起程度（0、1、2、3）；下蹲和站立（0、1、2、3）

（3）　实验室检查：

血常规：红细胞计数、血红蛋白、白细胞计数、血小板计数、中性粒细胞绝对值、淋巴细胞绝对值

尿常规：尿蛋白、尿红细胞、尿白细胞

肝功能：丙氨酸氨基转移酶（ALT）、天门冬氨酸氨基转移酶（AST）、白蛋白（ALB）

肾功能：尿素氮（BUN）、肌酐（CRE）

血糖

血沉（ESR）

CRP

（4）　药物治疗；

（5）　记录合并用药；

（6）　记录不良事件；

（7）　预约下次访视时间；

疗效评价

7.1 主要疗效指标

治疗 12、24 周的 PMR-AS<10 的比率。

7.2 次要疗效指标

12、24 周的 PMR-AS，CRP，ESR

7.3 疗效评定标准

PMR-AS<10：

（1）提示病情明显改善，低疾病活动度

（2）否则，视为未达到缓解；

8. 安全性评价

8.1 不良事件

记录整个试验过程中受试者发生的任何未预期或不适的症状、体征、疾病或可能导致身体伤害，暂时与药物有关联，但不一定与药物有因果关系的事件。记录不良反应时应注明该不良反应是否与试验药物有关；以及是否与风湿性多肌痛有关。

可能的不良反应：轻度上呼吸道感染、轻度泌尿道感染、带状疱疹，上述症

状发生率 2%左右。对应的处理措施见 10.12

8.2 实验室检查

于研究过程中进行实验室检查，内容如下：

血常规：红细胞计数、血红蛋白、白细胞计数、血小板计数、中性粒细胞绝对值、淋巴细胞绝对值

尿常规：尿蛋白、尿红细胞、尿白细胞

肝功能：丙氨酸氨基转移酶（ALT）、天门冬氨酸氨基转移酶（AST）、白蛋白（ALB）

肾功能：尿素氮（BUN）、肌酐（CRE）

血糖

凝血指标：凝血酶原时间（PT）、活化部分凝血活酶时间（APTT）、纤维蛋白原（FIB）

研究开始前需获得相关实验室检查项目的正常值范围。受试者如在用药后，实验室检查结果明显异常的应访视至正常范围（或回到基线状态）。如果在一定时期内，未能恢复正常（或回到基线状态），研究者应进行病因学检查和诊断。

9.　伴随疾病和治疗

9.1　伴随疾病

在知情同意书给予时出现的疾病将被认为是伴随疾病，将被记录在病例报告表上。

9.2　伴随治疗

所有在受试者进入研究时或研究期间任何时间进行的其它治疗被认为是伴随治疗，所用药品以通用名的形式记录在病例报告表中。试验期间的伴随用药必须是受试者所必需使用的，研究者若认为对试验药物无干扰作用方可酌情决定是否给予，剂量应保持在最低水平。

因某些疾病不得不在试验期间使用时，除符合以上原则，应注意对给药剂量的控制。

9.3　禁用药物

研究期间任何时间开始使用禁用药物或治疗的受试者将被判定为研究治疗失

败并且被剔除出本研究。研究期间任何时间禁止使用下列药物和治疗：

（1） 其他试验性药物（生物或非生物制剂）。试验性药物包括在本国未批准销售但正在使用的任何药物；

（2） 同时入组另一项试验性药物的临床研究或可能干扰本研究试验的非药物治疗的临床研究；

（3） 白介素-6 治疗（例如，雅美罗）；

（4） 其他生物制剂（利妥昔单抗）；

（5） 禁用活疫苗。

10. 不良事件及严重不良事件

研究者有责任发现并记录符合本研究方案规定的不良事件和严重不良事件定义的事件。

10.1 不良事件的定义

不良事件是指发生在使用某种药物的患者或临床研究对象身上的任何一件不利的医疗事件，它与此种治疗不一定有因果关系。

因此不良事件可以是任何不利的和未预料到的体征（包括有临床意义的实验室检查结果异常）、症状或与应用药物暂时相伴的疾病（新出现的或原有疾病的恶化）；如果是市售药品，还应包括使用后未产生预期疗效（即缺乏疗效）、滥用和误用。

不良反应包括：

（1）试验药具有良好的耐受性，不良反应发生率极低，若有过敏反应时，可按一般抗过敏处理方法，给予抗组胺药或糖皮质激素等及时对症治疗；

（2）原有疾病出现明显的或未预见的加重或恶化；

（3）原有的慢性疾病恶化或间断发作性疾病加重，表现为频率和/或强度增加；

（4）在使用研究用药后发现或诊断的病情，即使它在研究开始前可能已经存在；

（5）怀疑相互作用而出现的体征、症状或临床后遗症；

（6）与研究用药过量或其它合并药物过量相关的体征、症状或后遗症（受试者自行过量用药除外）；

（7）与研究用药过敏或毒性相关产生的反应；

（8）预期的药理学或生物学反应明显失败。

不良事件不包括：

（1）内科和外科检查治疗过程（如内镜检查、阑尾切除术），但导致需作这些检查的疾病是不良事件；

（2）有害的医疗事件并未发生的情况（如因社会收容或方便病人而收入院）；

（3）在研究开始时存在或发现的原有疾病或病情出现预期的周期性波动、但并未恶化；

（4）正进行研究的疾病或紊乱，或者与疾病或紊乱相伴的预期进展、症状或体征，除非它们比受试者所患疾病的预期更为严重。

10.2 严重不良事件的定义

严重不良事件是指发生于任一剂量水平上的符合以下任何条件的不良事件：

（1）导致死亡

（2）危及生命

注：危及生命是指此事件出现时受试者有死亡的危险。这个定义不包括如果病情进一步加重后可能导致死亡的事件。

（3）入院治疗或住院时间延长

注：一般而言，“住院”是指受试者不适合在门诊或急诊观察或处理，而需正式入院或急诊留观（通常至少要过夜）。住院期间出现的合并症为不良事件。如果因合并症导致住院时间延长或达到其它任何严重不良事件的标准，也属于严重不良事件。如果因为既往疾病而进行的择期手术且此疾患较基线时未加重，则不属于严重不良事件。

（4）残疾

注：残疾是指某人正常生活能力的实质性丧失，不包括有关的细小的病症：如单纯性头痛、恶心、呕吐、腹泻、流行性感冒、意外损伤（如踝关节扭伤）等，尽管它们可能对日常生活能力有一定影响，但并不是实质的（长期）丧失。

（5）先天畸形/异常

（6）某些需要医学或科学的判断决定在此种情形下是否紧急报告的情况：虽不会导致死亡、立刻危及生命或住院治疗但对受试者可能造成危害、或可能需要内科或外科治疗以阻止上述定义所列后果之一发生的重要的医疗事件，则可认为是一种严重不良事件。此种医疗事件包括浸润性或恶性癌肿、需在急诊室或家中给予重症监护的过敏性支气管痉挛、尚未导致住院治疗的血恶液

质或惊厥、以及药物依赖或药物滥用的事件。

10.3 缺乏疗效

缺乏疗效本身不能作为不良事件报告，任何缺乏疗效导致的症状/体征或后遗症只有符合不良事件/严重不良事件标准，才需报告。

10.4 临床检验结果异常和其它异常指标作为不良事件或严重不良事件

某些异常的实验室检查结果（如临床生化、血液学、尿液分析）或其它异常指标（如心电图、生命体征等）经研究者判定具有临床意义，如果它们符合 10.1 部分不良事件的定义（“不良事件的定义”），或 10.2 部分的严重不良事件定义（“严重不良事件的定义”），则必须将其记录为不良事件或严重不良事件予以记录。在用药后发现的，或者在基线评估时存在并在研究开始后加重的有临床意义的实验室检查结果异常及其它异常所见，则应作为不良事件或严重不良事件。但是，与所研究疾病有关的有临床意义的异常实验室检查结果或其它异常所见，除非研究者判定较所预料的受试者的病情更为严重，否则不包括在不良事件或严重不良事件中。在研究开始时存在或被发现但未加重的异常实验室检查结果或其它异常所见，也不包括在不良事件和严重不良事件中。

由研究者来决定一项异常实验室检查结果或其它异常所见是否具有临床意义。

10.5 发现不良事件的方法

不良事件将通过口头提问的方式获悉并记录于 CRF 不良事件页。研究者或指定人员每次询问不良事件时应使用相同的问题，以免造成受试者间的差异。研究者可以问：

“上次访视以来你是否感觉有什么不同？”

针对受试者对上述提问的回答，研究者可根据患者特殊主诉再提些相关问题，如：

不适症状有多严重？

多长时间出现一次？

一般症状会持续多久？

研究者还需询问受试者关于以往未解决的不良事件的情况。研究者要评估不良事件的强度、严重程度、与研究药物的相关性以及处理措施。

从试验开始前至试验结束或相应的退出访视，研究者均要询问受试者上述问

题。

10.6 不良事件和严重不良事件的记录

发生不良事件/严重不良事件时，研究者有责任回顾所有相关记录（如病程记录、实验室检查和诊断报告），并将与事件有关的资料记录于受试者的病例报告表中。

记录不良反应时应注明该不良反应是否与试验药物有关；以及是否与风湿性多肌痛有关。研究者应尽量依据症状、体征和/或其它临床资料就事件作出判断。在此情况下，诊断应作为不良事件和/或严重不良事件进行记录，而不是受试者的体征/症状。

10.7 不良事件和严重不良事件的评估

10.7.1 严重程度的判定

在研究期间，研究者应依据自己的临床判断，对报告的每起不良事件和严重不良事件的严重程度作出评估。记录在病例报告表中不良事件和严重不良事件应该按下列标准归类：

轻度：只引起受试者轻微不适，不影响日常活动，受试者比较容易耐受的事件；

中度：引起受试者明显不适并妨碍了正常日常活动的事件；

重度：导致无法进行正常日常活动的事件。

注意不要混淆重度的不良事件与严重不良事件：重度是用来衡量事件严重程度的一个类别，不良事件和严重不良事件都可以评为重度，凡符合 10.2 中“严重不良事件”定义的事件，都应列入严重不良事件。

10.7.2 因果关系的判断

研究者必须依据临床经验判断每起不良事件/严重不良事件与所研究药物之间的关系。按与药物肯定有关、很可能有关、可能有关、可能无关、无关五级来评定不良事件与试验药物之间的关系。

（1）肯定有关：用药及反应发生时间顺序合理；停药反应停止，或迅速减轻或好转；再次使用，反应再现；同时有文献资料佐证；并已排除原患疾病等其他混杂因素的影响；

（2）很可能有关：无重复用药史，其余同“肯定”或虽然有合并用药，但基本可排除合并用药导致反应发生的可能；

（3）可能有关：用药与反应发生时间关系密切，同时有文献资料佐证；但引发不良反应的药品不止一种，或原患疾病病情进展因素不能除外；

（4）可能无关：不良反应与用药时间关系不密切，反应表现与已知该药的不良反应不相吻合，

原患疾病发展同样可能有类似临床表现；

（5）无关：不良反应与用药时间之间没有关系。

不良反应是指 1、2 和 3 之和。

其它原因，如潜在疾病的自然病史、同时进行的其它治疗、危险因素以及暂时与所研究药物有关的事件均需考虑和检查。研究者还应查阅临床研究者手册和/或研究药物（如为市售药品）的相关资料用以作出评估。

研究者在最初报告出现某种严重不良事件时，可能只有很少的信息，然而，在向上呈报严重不良事件的 CRF 之前，研究者对每一事件进行因果关系评估十分重要。研究者可以根据访视资料更改因果关系的判断并对相应的 CRF 页进行相应修改。因果关系的判断是必须的指标之一。

研究者应依照 CRF 中 SAE 表的格式提供因果关系评估。

10.8 不良事件和严重不良事件的访视

一旦报告发生不良事件/严重不良事件，研究者应对每一个受试者进行访视。对所有在前次访视/复诊中已经记录的并持续存在的不良事件/严重不良事件，应在此次的访视/复诊中进行复查。

所有不良事件/严重不良事件都必须访视至其缓解、病情稳定、事件原因另有其它解释或受试者失访。问题一旦得到解决，CRF 中不良事件/严重不良事件部分应得到及时更新。研究者可以在访视中增加另外的一些检查，这可能有助于阐明不良事件或严重不良事件的本质和/或原因。这可能包括另外的实验室检查或研究、病理学检查或其它专业人士的会诊。

新的或更新后的资料应被记录于初始完成的 CRF 严重不良事件页上，研究者应对新的或更新后的资料签名并注明日期。

10.9 严重不良事件的处理和报告

研究者一旦意识到受试者发生了严重不良事件，必须立即采取治疗措施。严重不良事件报告表应尽可能完整、详细地记录所有能得到的与事件有关的资料。在初始报告中，研究者就应依据 10.7.2（因果关系的判断）进行因果关系的评估，

并向伦理委员会汇报。

报告机构	电话	传真	主要联系人
浙江大学医学院附属第一医院	**13906539996**	**0571-87235614**	林进

10.10　研究结束后的不良事件和严重不良事件

研究结束后的不良事件/严重不良事件是指任何发生在 10.5 中（发现不良事件和严重不良事件的方法、频率和时限）规定的不良事件/严重不良事件访视期限以外的任何事件。研究者不必主动探求发生在已结束临床研究和访视的受试者身上的不良事件和严重不良事件。但是，如果研究者在一名受试者已脱离研究后的任何时候知悉了发生了任何严重不良事件（包括死亡），而且此事件有可能与研究用药有关，研究者应联系受试者并做相应处理，并做相应记录，并向伦理委员会汇报。

10.11 与参加试验有关的不良事件和严重不良事件

被认为与参加研究有关的严重不良事件（如操作、创伤性检查，现有治疗方案的改变），无论它发生在治疗前还是治疗后，都应及时处理并记录，并向伦理委员会汇报。

10.12 本研究不良反应及处理方法

1. 血常规和肝功异常：

	继续原方案并给予对症治疗	托法替布剂减量并给予对症治疗	停托法替布
血常规	白细胞计数≥3×10^9/L	3×10^9/L>WBC>2.5×10^9/L	白细胞计数<2.5×10^9/L
肝功	转氨酶升高<1.5 倍	1.5 倍≤转氨酶升高<3 倍	转氨酶升高≥3 倍

上述情况可酌情加用保肝药和升白药。研究者需根据出现的不良事件与药物的相关性，对用药方案进行调整。如复查肝功能正常，可恢复原治疗剂量；若再次出现肝功能异常，减量/停药后，不再加量。

连续停用研究药物 8 周，患者退出研究。

2. 药物过敏：退出研究，根据临床经验治疗。

3. 轻度上呼吸道感染、轻度泌尿道感染、带状疱疹等其他不良事件：按临床诊疗常规进行诊治，可控可治。

如果出现严重不良事件应退出研究。

11. 用药依从性

在试验结束后，对患者的依从性进行评价，记录使用的药物数量，记录患者是否使用试验药物。

12. 退出研究

12.1 受试者完成试验

受试者按照试验要求完成计划安排的每次访视规定的所有内容，则视为完成试验。

12.2 受试者退出试验

任何进入试验的受试者（已签署知情同意书）由于任何原因未能按照上述定义的要求完成试验, 将被视为退出试验，不论其是否使用试验药物。

预计将随机入选 60 名患者，参加试验的受试者将获得唯一的随机号。

每个受试者均可在研究的任何阶段、任何理由（特殊或非特殊）退出该项研究而不受到歧视。患者的治疗将不会受到限制，在适当的情况下，研究者将使用常规的治疗方法为患者治疗。

应尽可能的访视退出试验的患者。随机后退出的患者在计划访视时退出应完成当次访视。所有随机后退出试验的受试者，均应尽量完成访视 3 要求的所有评估。

参加随机的所有受试者的病例报告表均要填写完整，并且在病例报告表的“试验总结”上记录所有被随机但未完成试验的受试者退出原因。此外，在“试验完成情况总结”上必须记录提前退出试验的受试者末次用药日期。

12.3 筛选和基线评估失败

患者在随机之前退出试验视为筛选失败。退出试验的原因需记录在患者筛选入选表中。

13. 数据管理

（1）研究者根据受试者的原始观察记录，将数据及时、完整、正确、清晰地载入病例报告表。

（2）确认所有病例报告表填写正确完整，并与原始资料一致。如有错误和遗漏，及时要求研究者改正。修改时需保持原有记录清晰可见，改正处需经研究者签名并注明日期。

（3）对于完成的病例报告表在研究者、数据管理员之间的传送应有专门的记录，

收到时应有相应的签名，记录需妥善保存。

（4）数据管理员在数据录入前再次核查，发现问题及时通知研究者，要求研究者作出回答。他们之间的各种疑问及解答的交换应当采用疑问表形式，疑问表应保存备查。

（5）数据管理员在进行数据录入前，要了解观察表格各项目的内容及编码情况，将编码工作过程记录于编码本保存。数据库命名应规范、易读、易查找。并保证其正确、安全和保密。

（6）数据录入员录入数据采用二次录入。录入过程发现问题或意外情况，应做好登记并及时报告，以便迅速处理问题，数据录入结束后应抽查部分观察表格，了解录入质量，分析并处理存在的问题。

（7）数据管理员应与主要研究者一起，按病例报告表中各指标数值的范围和相互关系拟定数据范围检查和逻辑检查内容。并编写相应的计算机程序，在输入前控制错误数据输入，找出错误原因加以改正，所有错误内容及修改结果应有记录并妥善保存。

（8）原始病例报告表在按要求完成数据录入和核查后，按编号的顺序归档保存，并填有检索目录等，以备查考。电子数据文件包括数据库、检查程序、分析程序、分析结果、编码本和说明文件等，应分类保存，并有多个备份保存于不同磁盘或记录介质上，妥善保存，防止损坏。所有原始档案应按我国《药物临床试验质量管理规范》的规定期限保存。

14. 统计分析

根据数据特征定稿，本方案提供统计学常规要求。

14.1 样本量估算

从有效性角度，根据统计学要求，采用差异性检验，采用双侧检验$\alpha = 0.05$，$\beta = 0.10$(把握度 90%)，以 12、24 周 CR 率为主要终点，预计试验组患者的 CR 率为 95%，对照组患者的 CR 率为 85%，优效/非劣效界值为 0.1，两组样本分配比例为 1:1，采 PASS11.0.7 软件计算，每组 38 例，同时考虑 18-20%的脱落，每组至少需要 47 例。最低有效总样本量 94 例, 试验组和对照组各 47 例。

14.2 分析数据集

（1）符合方案集（PPS）：指符合纳入标准、不符合排除标准、完成治疗方案的

病例集合，即对符合试验方案、依从性好、完成 CRF 规定填写内容的病例进行分析（PP 分析）。PP 分析主要用于主要疗效指标。

（2）安全数据集（SS）：至少接受一次治疗，且有安全性指标记录的实际数据。安全性缺失值不得结转；纳入可作评价的部分剔除病例，如年龄超过纳入标准的病例，但不包括使用禁用药物导致无法作安全性判断的病例。不良反应的发生率以安全集的病例数作为分母。

14.3 统计方法与内容

14.3.1 病例入组分析

- 列出总体入选及完成病例数，确定二个分析数据集（PPS，SS）。
- 列出脱落与剔除病例及其原因。

14.3.2 人口学资料及基线分析

描述性统计人口学资料及其他基线特征值：

- 连续变量计算其例数、均值、标准差、中位数、最小值和最大值。
- 计数和等级资料计算频数及构成比。
- 推断性统计结果（P 值）作为描述性结果列出。

14.3.3 疗效分析

疗效指标分析

- 治疗12、24周 PMR-AS<10的患者比率的比较，采用费希尔精确检验分析组间差异。
- 治疗12、24周的 PMR-AS 评分、CRP，ESR，采用重复测量方差分析比较组间差异。

14.4 安全性分析

- 计算不良事件和不良反应发生率；
- 计算严重不良事件发生率。
- 分系统列出不良事件和不良反应发生的频率和频数，计算百分比, 采用费希尔精确检验分析；
- 列出不良事件病例的详细列表；
- 列出不良反应病例的详细列表；
- 实验室检查、心电图、体检在试验后“正常转异常”或“异常加剧”的例数和转异率；

- 列出实验室检查、心电图、体检异常病例和临床解释。

14.5 统计软件与一般要求

- 采用 SPSS 18 软件分析；
- 所有的统计检验均采用双侧检验，P 值小于或等于 0.05 将被认为所检验的差别有统计学意义；
- 详细的统计方法将在统计分析计划中提供。

14.6 期中分析

本研究不进行期中分析。如试验中发生特殊情况，如疗效不确切或安全性问题，将由研究者和参与人员一同讨论决定。

15. 研究管理

15.1 遵从 GCP 的要求

应按照中国药品临床试验管理规范（GCP）的要求来进行本试验。

15.2 保护受试者的隐私权

在填写和管理病例报告表时，应考虑到保护患者的隐私，如以患者的随机号来代表患者等，同时可将患者名字的缩写当作其名字的代称。

15.3 知情同意书

在开始本试验前，试验人员必须将受试者知情同意书以患者能理解的方式向每一位参加本试验的患者予以解释说明，并获得患者自愿参加本项试验的书面形式的知情同意书。在 CRF 中填写获得书面知情同意书的日期。

（1）研究的性质和目的；

（2）研究过程；

（3）受益和风险；

（4）其他的替代治疗；

（5）受试者权利：患者拒绝参加本项试验或在试验进展的任何时候都可以退出本试验，患者的权益不会受到任何影响；

（6）保密协定；

（7）患者必须遵守的事项；

（8）以及认为可以保护患者权益的任何事项。

15.4 方案的修订程序

当必须修改方案或暂停本项试验时，主要研究者应立即将详细情况及原因通知所有参加试验的人员。如果是重大修改，应取得伦理委员会批准后实施。

15.5 病例报告表记录及修改程序

真实、准确的记录试验数据。

如需做任何修改，请保持修改前的记录清晰可辨，并署明修改日期。如修改范围较大或改动较大，则修改者应注明修改日期及修改的理由，同时签名。

15.6 质量控制和质量保证

（1）采用标准操作规程，以保证临床试验的质量控制和质量保证系统的实施。

（2）临床试验中所有观察到的结果和异常发现，均应及时加以认真核实、记录，保证数据的可靠性。临床试验中各种检查项目所使用的各种仪器、设备、试剂、标准品等，均应有严格的质量标准，并确保是在正常状态下工作。临床数据的记录和转移，必须由有经验的医师负责，并有专人监督或核对，以保证数据的科学性和准确性。临床试验的各种结论，必须来源于原始数据。

（3）负责试验的医师，应完整、详细、准确、及时地填写病例报告表（CRF）。交上级医师签名确认后按规定程序报送或保存。所有与试验有关的数据资料应集中管理与分析。

（4）建立数据保管、数据传递、数据查询的程序。保管的资料包括：受试者的研究病历、影像学资料、CRF、药品使用登记表、受试者筛选表、受试者鉴认代码表、严重不良事件报告表、各医院需填报的 GCP 表格、访视报告表及有关的各种原始医疗文件等。传递的数据包括：受试者总随机表、CRF、严重不良事件报告表及总结资料需使用的数据和资料。

（5）总结和分析临床试验结果时，必须采用规范的统计学分析方法，并请熟悉生物统计学的人员参与。

15.7 资料保存

（1）原始资料由研究单位保管。

（2）保存期至试验结束后 5 年。

（3）本次临床试验的所有资料，所有权属于浙江大学医学院附属第一医院。

15.8 项目进度计划

因为该疾病发病率不高，为了收集齐患者数目，研究的时间定为 2 年。

16. 论文发表

研究者及研究中心其他工作人员对由浙江大学医学院附属第一医院提供的所有资料和在研究过程中由参与研究的中心产生的所有数据（受试者的医疗记录除外）应予以保密。研究者或中心的其他工作人员除了用于此研究外不得将资料、数据或记录用于其它目的。这些限制不适用于：（1）不是因研究者或研究中心工作人员的错误致资料已公开发表；（2）出于取信于学术委员会或伦理委员会以评估此研究的目的必须予以公开的资料；（3）为了给参加研究的受试者提供适当的医疗保健必须予以公开的资料。

17. 参考文献

[1] 国家食品药品监督管理局. 药物临床试验质量管理规范（GCP2003 版）.

[2] 新药（西药）临床研究指导原则.卫生部药政局（1993 版）.

附表一　1982 年 Chuang 风湿性多肌痛诊断标准

1	年龄 50 岁以上
2	双侧疼痛和僵硬至少一个月，累及下列部位至少 2 处：颈部或躯干、肩或上肢近侧，髋部或大腿近端
3	血沉>40mm/h
4	排除巨细胞动脉炎以外的其他疾病

符合所有表现，才可诊断风湿性多肌痛。

附表二　2012 年 EULAR/ACR 风湿性多肌痛的分类标准

必需标准:	年龄＞ 50 岁，双肩痛，C反应蛋白和/或血沉异常

	无超声得分（0-6）	含超声得分（0-8）
晨僵＞ 45 min	2	2
髋部疼痛或运动受限	1	1
RF 及抗环瓜氨酸肽抗体阴性	2	2
其余关节无受累	1	1
至少有一侧肩部三角肌下黏液囊炎，　肱二头肌腱鞘炎，或肩关节滑膜炎;至少一侧髋部滑膜炎或转子处黏液囊炎	/	1
双侧肩三角肌下黏液囊炎，肱二头肌腱鞘炎，或肩关节滑膜炎	/	1

总分无超声评分，4分以上，诊断PMR；总分含超声评分，5分以上，诊断PMR

附表三 风湿性多肌痛疾病活动度(PMR-AS)评分

CRP (mg/dl)	
患者自我评价（0-10 可视量表）<br>0=无疼痛，10=疼痛无发忍受	
医生总体评价（0-10 可视量表）<br>0=无疼痛，10=疼痛无发忍受	
晨僵时间 min x0.1	
双肩关节（0-3）<br>0=抬起超过肩带，1=到肩带，2=低于肩带，3=不能抬起	
总计	

超过 10 分提示疾病缓解或处于低疾病活动

因臀部肌肉受累导致下蹲和站立困难，评分，
0=无困难，1=借助他人或上肢力量能下蹲和站立，2=借助他人或上肢力量能下蹲，但不能站立，3=不能下蹲和站立。

浙江大学医学院附属第一医院临床研究伦理委员会IIT伦理审查小组伦理审查批准件

Approval Letter of Clinical Research Ethics Committee of the First Affiliated Hospital, College of Medicine, Zhejiang University

受理号：IIT20200070C-R1　　　　批件号Reference Number：快审第（402）号

项目名称 Study Title	托法替布对风湿性多肌痛的治疗疗效和安全性前瞻性研究<br>Prospective study on the efficacy and safety of tofacitinib in the treatment of rheumatic polymyalgia		
组长单位 Lead Site	浙江大学医学院附属第一医院<br>First Affiliated Hospital, College of Medicine, Zhejiang University	科室 Department	风湿免疫科<br>Division of Rheumatology and Immunology
主要研究者 PI	林进<br>Jin Lin	协调研究者 SubI	陈伟钱<br>Weiqian Chen
审查文件 Documents Revie	见附件		

审查意见 Evaluation Comments:

☑ 批准Approval　☐ 修改后批准Conditional Approval　☐ 修改后再审Re-submission

☐ 暂停或者终止研究Suspended or Termination　☐ 不批准Disapproval

临床研究伦理委员会IIT伦理审查小组

主任或副主任签名Signature of Chairman:

日期Date：2020-08-05

浙江大学医学院附属第一医院临床研究伦理委员会（盖章）

Clinical Research Ethics Committee of the First Affiliated Hospital, College of Medicine, Zhejiang

备注:

1. 研究者应遵循伦理委员会批准的方案执行，实施过程应符合赫尔辛基宣言的原则。
2. 在试验实施过程中，对研究方案和知情同意等相关文件所作的任何修改，均需得到伦理委员会审查同意后方可实施。
3. 发生严重不良事件及可能影响风险受益比的任何事件和新信息须及时报告本院伦理委员会。
4. 接受伦理委员会持续审查的项目，请在到期前1个月（无论试验开始与否）提出再次审查的申请。
5. 如有不依从/违背方案或暂停/提前终止的试验项目，应及时以书面文件报告本院伦理委员会；临床试验结束后，须及时向伦理委员会提交结题报告。
6. 本批件有效期1年（自批准之日起），如试验逾期未实施即自行废止。
