# Supplementary figures and images for: Efficacy and Safety of Tofacitinib in Patients with Polymyalgia Rheumatica (EAST PMR): An open-label randomized controlled trial
Source: PLoS Med. 2023 Jun 29;20(6):e1004249. doi: 10.1371/journal.pmed.1004249 (PMC10309604; doi:10.1371/journal.pmed.1004249)

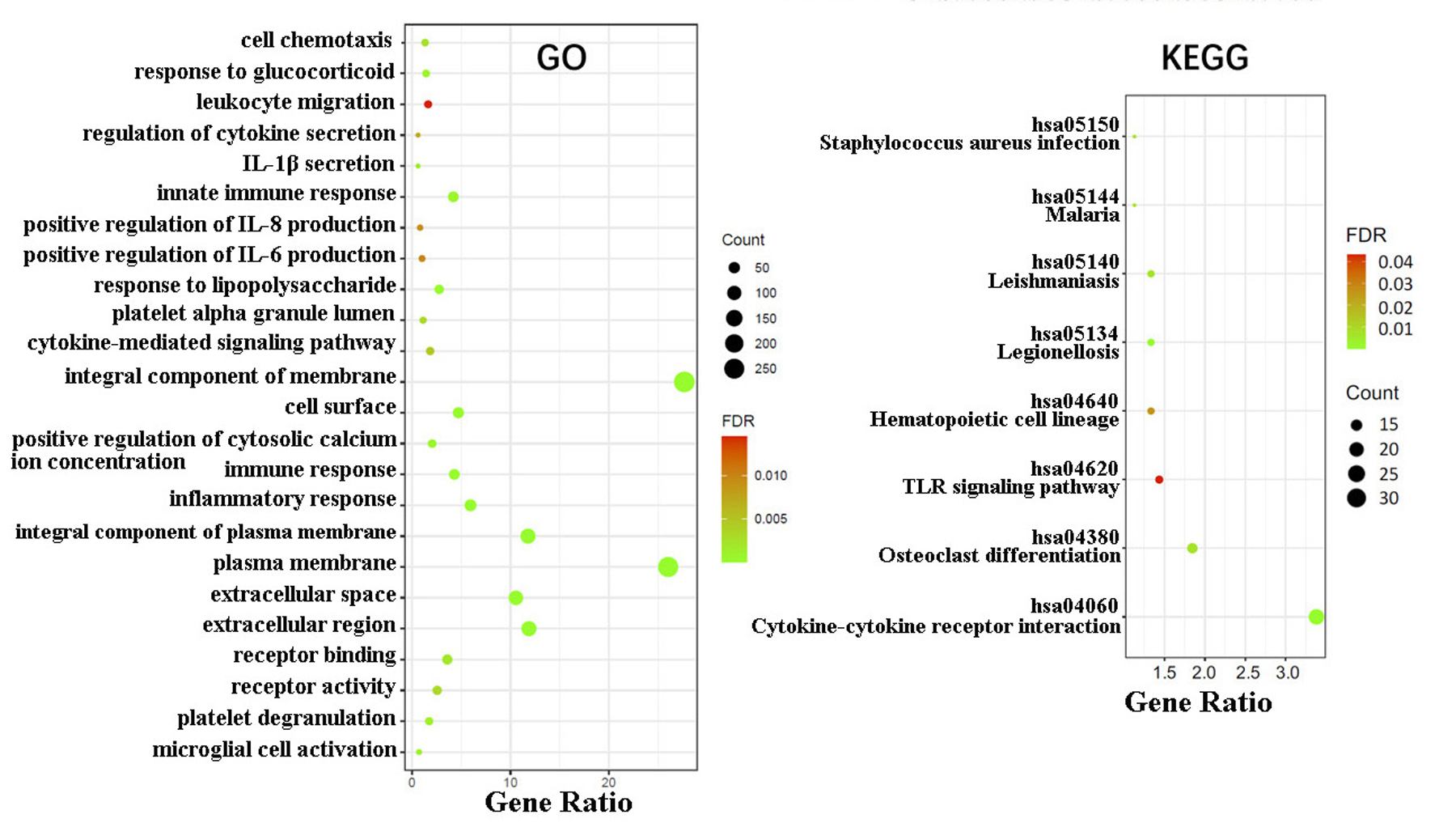

Supplement: S1 Fig — (JPG) [file pmed.1004249.s003.jpg]

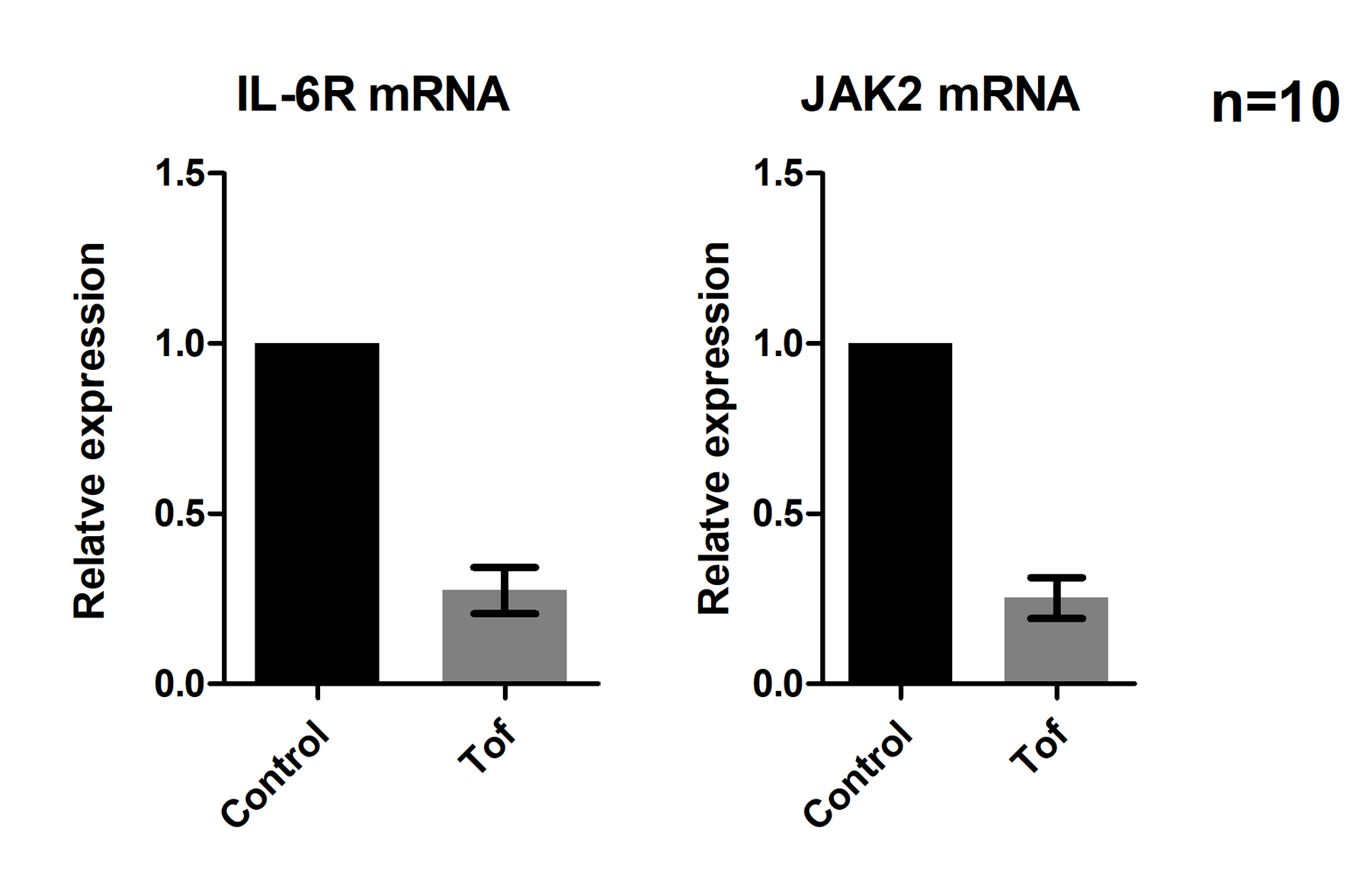

Supplement: S2 Fig — (TIF) [file pmed.1004249.s004.tif]

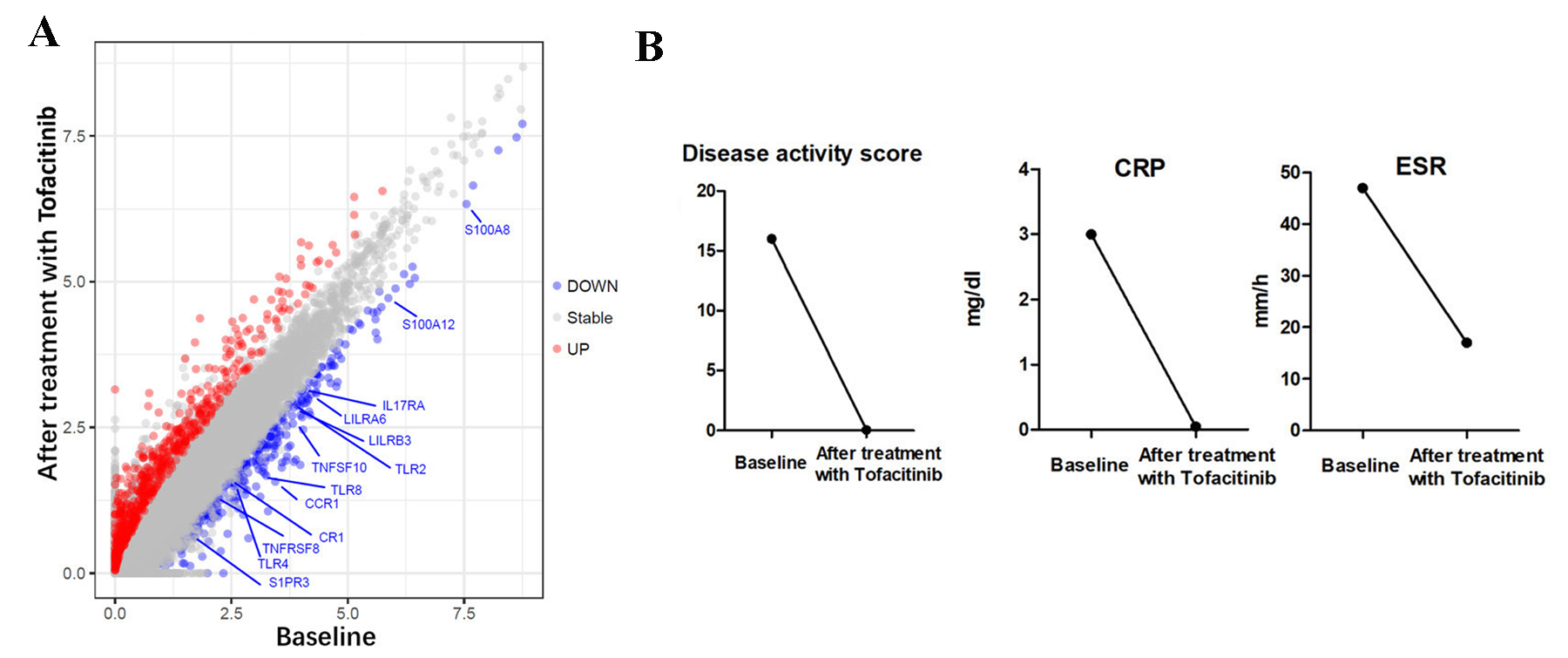

Supplement: S3 Fig — (TIF) [file pmed.1004249.s005.tif]

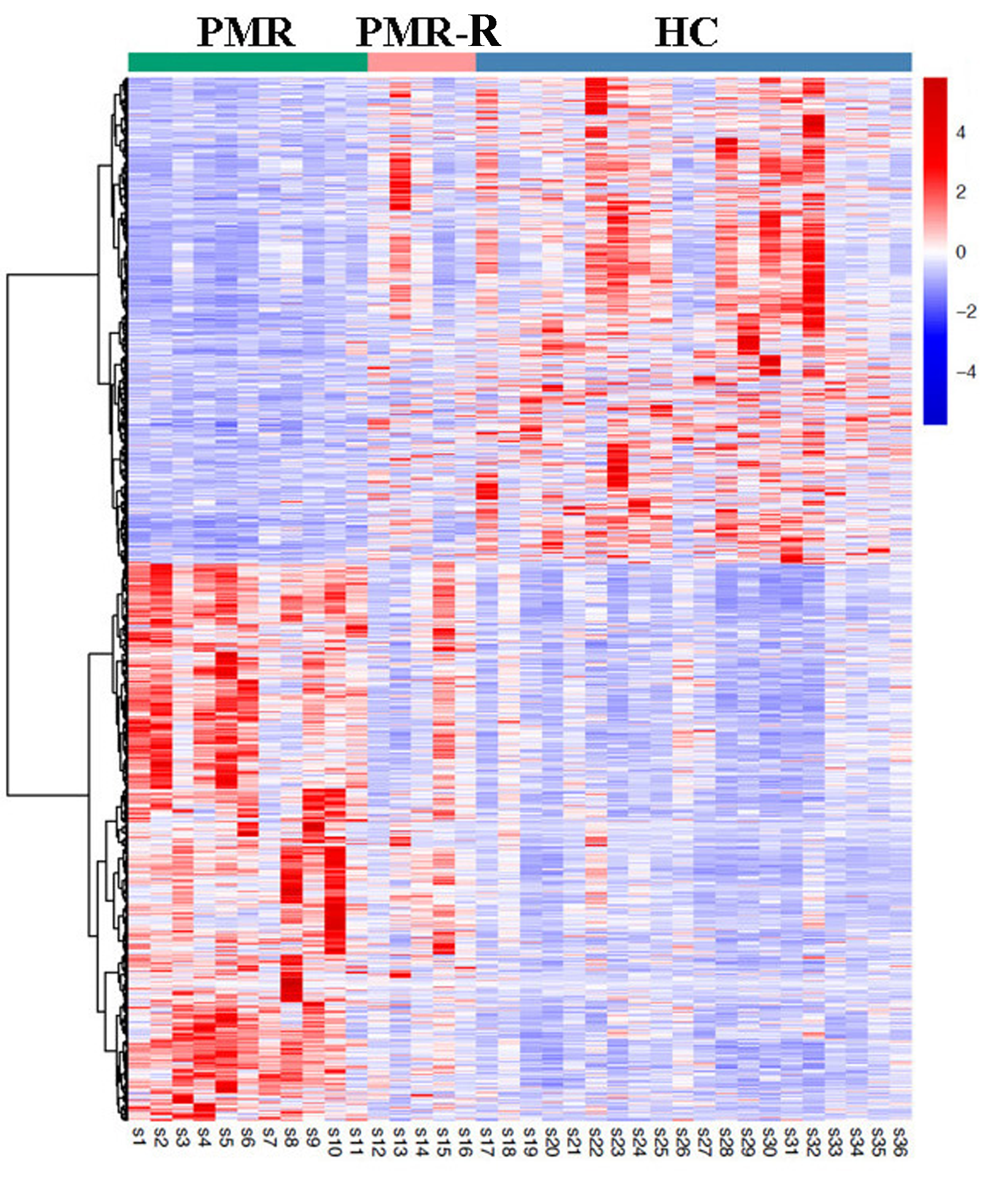

Supplement: S4 Fig — (TIF) [file pmed.1004249.s006.tif]
