# Supplementary material for: Efficacy and Safety of Tofacitinib in Patients with Polymyalgia Rheumatica (EAST PMR): An open-label randomized controlled trial
Source: PLoS Med. 2023 Jun 29;20(6):e1004249. doi: 10.1371/journal.pmed.1004249 (PMC10309604; doi:10.1371/journal.pmed.1004249)
Supplement: S1 Table — (DOCX) [file pmed.1004249.s007.docx]

IL6R Forward: 5'-CCCCTCAGCAATGTTGTTTGT-3'

Reverse: 5'-CTCCGGGACTGCTAACTGG-3'

IL1B Forward: 5'-ATGATGGCTTATTACAGTGGCAA-3'

Reverse: 5'-GTCGGAGATTCGTAGCTGGA-3'

Interleukin 1 receptor type 1 (IL1R1)

Forward: 5'-ATGAAATTGATGTTCGTCCCTGT-3'

Reverse: 5'-ACCACGCAATAGTAATGTCCTG-3'

Janus kinase (JAK) 2

Forward: 5'-TCTGGGGAGTATGTTGCAGAA-3'

Reverse: 5'-AGACATGGTTGGGTGGATACC-3'

Toll Like Receptor 2 (TLR2)

Forward: 5'-ATCCTCCAATCAGGCTTCTCT-3'

Reverse: 5'-GGACAGGTCAAGGCTTTTTACA-3'

TLR4 Forward: 5'-AGACCTGTCCCTGAACCCTAT-3'

Reverse: 5'-CGATGGACTTCTAAACCAGCCA-3'

TLR8 Forward: 5'-ATGTTCCTTCAGTCGTCAATGC-3'

Reverse: 5'-TTGCTGCACTCTGCAATAACT-3'

Interleukin 17 Receptor A (IL17RA)

Forward: 5'-GCTTCACCCTGTGGAACGAAT-3'

Reverse: 5'-TATGTGGTGCATGTGCTCAAA-3'

Complement receptor 1 (CR1)

Forward: 5'-AGAGGGACGAGCTTCGACC-3'

Reverse: 5'-TCAGGACGGCATTCGTACTTT-3'

C-C motif chemokine receptor 1 (CCR1)

Forward: 5'-GACTATGACACGACCACAGAGT-3'

Reverse: 5'-CCAACCAGGCCAATGACAAATA-3'

S100 calcium-binding protein A8 (S100A8)

Forward: 5'-ATGCCGTCTACAGGGATGAC-3'

Reverse: 5'-ACTGAGGACACTCGGTCTCTA-3'

S100A12 Forward: 5'-AGCATCTGGAGGGAATTGTCA-3'

Reverse: 5'-GCAATGGCTACCAGGGATATGAA-3'

GAPDH Forward: 5'-GGAGCGAGATCCCTCCAAAAT-3'

Reverse: 5'-GGCTGTTGTCATACTTCTCATGG-3'
