# Supplementary material for: Efficacy and Safety of Tofacitinib in Patients with Polymyalgia Rheumatica (EAST PMR): An open-label randomized controlled trial
Source: PLoS Med. 2023 Jun 29;20(6):e1004249. doi: 10.1371/journal.pmed.1004249 (PMC10309604; doi:10.1371/journal.pmed.1004249)
Supplement: S2 Table — (DOCX) [file pmed.1004249.s008.docx]

**eTable 2** Demographic and clinical parameters of 11 PMR and 20 healthy controls in the first cohort

|  | PMR (*n* = 11) | Healthy controls (*n* =20) | *P* value |
| --- | --- | --- | --- |
| Age, mean ± SD years | 68.0±8.3 | 63.7±9.8 | 0.142 |
| Female/male | 10/1 | 17/3 | 0.67 |
| Disease duration, mean ± SD months | 10.4±14.8 | NA |  |
| Pain, VS score 0-10, mean ± SD | 4.9±2.2 | NA |  |
| Physician global assessment, VS score 0-10, mean ± SD | 4.7±2.9 | NA |  |
| Morning stiffness, min, mean ± SD | 17.3±21.8 | NA |  |
| EUL, mean ± SD | 1.0±0.8 | 0 |  |
| CRP, mean±SD mg/dl | 3.9±3.1 | 0.2±0.2 | <0.0001 |
| ESR, mean±SD mm/h | 67.1 ±24.9 | 10.5±5.2 | <0.0001 |
| PMR activity disease scores (PMR-AS), mean±SD | 16.3±7.3 | NA |  |

NA: not applicable, VS: visual analogue scale, EUL: ability to elevate the upper limbs; CRP: C-reactive protein; ESR: erythrocyte sedimentation rate.
